# Cortical network responses map onto data-driven features that capture visual semantics of movie fragments

**DOI:** 10.1038/s41598-020-68853-y

**Published:** 2020-07-21

**Authors:** Julia Berezutskaya, Zachary V. Freudenburg, Luca Ambrogioni, Umut Güçlü, Marcel A. J. van Gerven, Nick F. Ramsey

**Affiliations:** 1 Brain Center, Department of Neurology and Neurosurgery, University Medical Center Utrecht, Heidelberglaan 100, 3584 CX Utrecht, The Netherlands; 2 Donders Institute for Brain, Cognition and Behaviour, Radboud University, Montessorilaan 3, 6525 HR Nijmegen, The Netherlands

**Keywords:** Language, Perception, Neural encoding

## Abstract

Research on how the human brain extracts meaning from sensory input relies in principle on methodological reductionism. In the present study, we adopt a more holistic approach by modeling the cortical responses to semantic information that was extracted from the visual stream of a feature film, employing artificial neural network models. Advances in both computer vision and natural language processing were utilized to extract the semantic representations from the film by combining perceptual and linguistic information. We tested whether these representations were useful in studying the human brain data. To this end, we collected electrocorticography responses to a short movie from 37 subjects and fitted their cortical patterns across multiple regions using the semantic components extracted from film frames. We found that individual semantic components reflected fundamental semantic distinctions in the visual input, such as presence or absence of people, human movement, landscape scenes, human faces, etc. Moreover, each semantic component mapped onto a distinct functional cortical network involving high-level cognitive regions in occipitotemporal, frontal and parietal cortices. The present work demonstrates the potential of the data-driven methods from information processing fields to explain patterns of cortical responses, and contributes to the overall discussion about the encoding of high-level perceptual information in the human brain.

## Introduction

Semantic processing of audiovisual material is a topic of great interest in cognitive neuroscience. A lot of knowledge has been accrued with studies focusing on the visual object processing, object categorization and processing of various semantic attributes of the visually perceived objects^1–3^. We have come to understand a lot about the ventral stream of visual processing in the brain^4,5^, the object categorization ability of inferior temporal cortex^6–8^ as well as the specific roles of fusiform face area^9^, parahippocampal place area^10^, the motion processing temporal region^11^ and other areas involved in high-level visual processing^4,12–14^.

Most studies address the topic with a reductionist approach, with carefully constructed tasks and stimuli to investigate a particular aspect of brain function^15^. With new tools available to investigate high-dimensional phenomena, more holistic approaches become feasible. Complex brain mechanisms may be identified or characterized by mapping brain responses onto informational structures that are extracted from non-constrained sensory material. More concretely, one can utilize recent advances in computational modeling in different domains such as computer vision and natural language processing that are driven by extraction of high-level semantic relations directly from the unprocessed input, such as images^16,17^ and texts^18–20^. Among the most recent and most powerful such advances are deep artificial neural network models. Not only do these models achieve unprecedented performance on solving complex tasks (for example, visual object identification or text classification), but there is also evidence that these models are capable of making errors in semantic judgements that are similar to the errors humans make, thereby mimicking aspects of human cognition^21–23^.

Both of these factors contributed to an increasing interest towards these models in the cognitive neuroscience community. Many have investigated the internal representations extracted by artificial neural networks and used them to model, or ‘fit’, the brain activity associated with perception of images^24–28^, music^29^ and speech sounds^30–32^. Multiple studies showed that these models can fit evoked cortical responses better than alternative representations of stimuli typically based on hand-engineered filters for low-level perceptual input, for example, Gabor filters for pixel or sound spectrogram input^32–34^. Artificial neural network approaches to modeling language have also shown to correlate well with the cortical responses to language related stimuli^35–37^.

Yet many of these studies used stimuli that were constrained in one way or another to satisfy the specific question at hand. We think we can learn more about how the brain makes sense of large amounts of continuously perceived visual information if we examine brain signals in a natural context. In essence, here we present a case for feasibility, where we reduce the complexity of natural high-level visual material to key components that represent sematic information. This reduction is accomplished by mining visual information from the perceived input followed by modelling semantic and language information content. The extracted semantic components are used to model the cortical responses driven by high-level processing of perceptual information.

In the present study, we used a large dataset, in which participants watched a short feature film while their brain responses were recorded with electrocorticography (ECoG). The data were originally collected for language mapping in the patient population. These data were previously analyzed with respect to the auditory processing of the film soundtrack^30,32,38^. Our previous work also used functional magnetic resonance imaging (fMRI) recordings during the same task and in the same subjects. In the present study, we only focus on the ECoG data (due to a larger number of subjects available as well as its better temporal resolution and signal-to-noise ratio) and only on the visual aspect of the film. We passed the film frames first through a set of algorithms including an automatic visual concept recognition system and a language model to extract high-level semantic components. The first step provided us with a list of visual objects and concepts present in film frames and the second step enriched the extracted representation by incorporating semantic and linguistic ties between words corresponding to extracted visual concepts. The resulting set of key components were then imposed on the ECoG data to assess cortical representation thereof. We found that (1) the reduction of the visual data complexity yielded principal components that are readily interpretable in terms of semantic concepts, and (2) these components were represented in brain signals and distributed functional cortical networks. Our approach shows that processing of meaning in the visual stream of the film maps onto brain regions associated with corresponding functionality: lateral fusiform gyrus for processing faces, parietotemporal network for processing movement and lateral occipital complex for processing objects, scenes and landscape frames. We believe this approach has potential for deepening our understanding about how the human brain extracts meaning from visual information presented in an unconstrained natural context.

## Results

We investigated whether the visual semantic information obtained from a short film through a bottom-up computational approach could be informative in explaining the associated neural responses. First, we developed a semi-automatic bottom-up approach to extract principal *semantic* *components* that captured high-level conceptual information in the film’s visual stream. The extracted components proved to reflect fundamental semantic differences between film frames, such as presence or absence of people, motion versus still/wide-shot frames, human faces versus human bodies. Next, the semantic components were used to model the neural data collected from 37 subjects during a film-watching electrocorticography (ECoG) experiment. The model fit showed significant prediction accuracy peaking at 320 ms after frame onset primarily in occipitotemporal, parietal and inferior frontal cortices. Further analyses showed that the brain areas with significant prediction accuracy could be subdivided into distinct cortical networks, each engaged in processing of specific semantic components in the film’s visual stream of information.

### A semi-automatic bottom-up approach to obtain vectors of visual semantics from film frames

In order to extract semantic meaning, we combined the advances in various fields (deep learning, computer vision, natural language processing) to obtain visually driven semantic representations that can be used to study the neural responses. For this, we devised a pipeline that allowed us to combine recently published algorithms and obtain semantic representations.

**Figure 1:**
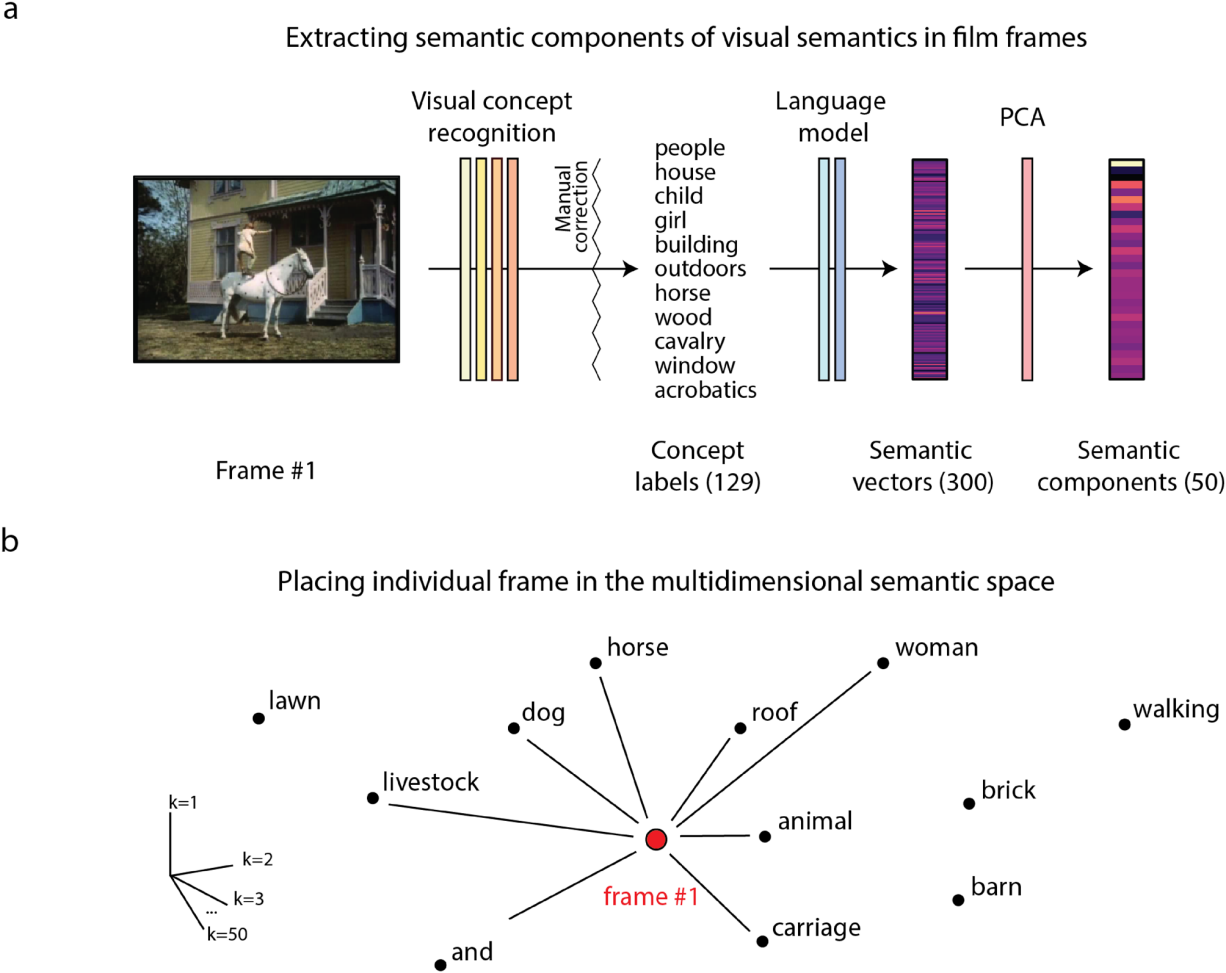
Extraction of semantic components from the visual stream of the film. (**a**) Semi-automatic pipeline for extraction of the semantic components. First, a film frame is passed through an automatic visual concept recognition system (*Clarifai*) to extract concept labels. Then, the extracted labels are passed through a language model (*fastText*) to obtain 300-dimensional semantic vectors, or word embeddings. The semantic vectors are averaged over all labels assigned to one frame, resulting in one averaged semantic vector per frame. The dimensionality of the vectors is further reduced by applying a principal component analysis to the averaged semantic vectors. The final result is a set of 50-dimensional semantic components that are used further to model the neural responses. (**b**) Example of how averaging of all concept labels per frame affects the semantic representation. Each word in the language model (*fastText*) can be seen as a point in a 300-dimensional semantic space. Neighboring words are assumed to capture similar semantics. Averaging in this space results in a new point that is placed in a neighborhood of all the words that are being averaged. Averaging has a capacity to represent combined complex meaning. In this example, the new point is in between the individual words ‘horse’, ‘carriage’ and ‘roof’, thus combining the meanings of these words together.

Our semi-automatic pipeline contained three stages: the visual concept recognition stage (I), the language model stage (II) and the dimensionality reduction stage (III, Fig. 1a). In stage I we employed an artificial neural network model called *Clarifai* (www.clarifai.com), a state-of-the-art commercial computer vision model, to obtain labels of the objects and concepts present in each frame. *Clarifai* processes raw pixel information and generates the most likely concept labels together with their probabilities. The outputs of this model are referred to as *concepts* rather than *objects* because the system is capable of recognizing not only physical items in an image but also emotions, qualities, actions and some abstract concepts. The network used a preset dictionary of 5,000 concept labels and generated 20 concept labels per image frame. Despite the overall remarkable quality of the concept recognition system, we performed a manual check on the extracted labels and adjusted all incorrect assignments. Therefore, we call our pipeline semi-automatic.

In principle, we could have used the extracted concept labels directly to model neural activity, as has been done before^39^. However, our intention was not to limit the study to a preselected number of concept or object labels. Instead, we aimed to exploit the semantic relationships between different labels, including those absent in the present stimulus material, such that similar labels would get similar representations and dissimilar labels would get dissimilar representations. To achieve this, we subsequently applied a language model that could enrich our concept space with complex semantic and language relations between concept words.

To some extent, capturing of semantics of the visually perceived concepts can be achieved through the co-occurrence of concept labels. However, the label co-occurrence could also lead to false associations. For example, the fact that the main character (Pippi) and her horse co-occur in many frames does not necessarily mean than they should have similar semantic representations. Likewise, the fact that another character (Mrs. Settergren) only appears in the breakfast scene does not mean she should be associated specifically with the meal. Moreover, use of a universal space of the language model renders the model more generalizable in that if one were to study a different film with a different set of present visual objects, one would be able to project them onto the same semantic space as the present film and compare the results.

To extract semantic vectors of each concept label in the frame (stage II of the pipeline), an external language model was applied, called *fastText*^40^ (www.fasttext.cc)*.* It is important to note, that we call it a language model here for simplicity and convenience, although strictly speaking here we only use the result of the language model training—the learned word embeddings, or semantic vectors. *FastText* is a shallow artificial neural network (*skip-gram* model) that has been trained on a large number of texts to extract word embeddings for each word in the language vocabulary based on the word’s context. The model has been shown to capture semantic relationships between words^20^. The idea behind the use of semantic vectors is not only to simplify computations but to create a multidimensional representational space where mathematical operations such as addition and multiplication should hold. We obtained semantic vectors for each concept in the frame using the pretrained *fastText* model, and averaged the vectors over all concepts in the frame. This resulted in one semantic vector of the visual information per frame. We also verified that projecting the averaged semantic vector to the *fastText* space resulted in a point that was between, and closest to, the multiple individual concept labels of the frame (see example in Fig. 1b).

The dimensionality of the data was further reduced to (1) represent the semantic space of our film more adequately (given that the concepts in the film likely covered only a small fraction of the entire semantic space of the *fastText* language model), and (2) determine the components of the most variance across the semantic vectors. To achieve this, we performed a principal component analysis on the previously obtained averaged semantic vectors (stage III). We found that projection to a space with just 50 principal semantic components accounted for 99.95% of all variance in the original averaged semantic vectors. Thus, from the averaged *fastText* semantic vectors per frame we obtained a smaller number of highly representable semantic components, ranked according to the amount of semantic variance they explained.

It is important to note that the extracted concept labels as well at the language word embedding model were based on English whereas the soundtrack of the film was in Dutch. However, we do not expect it to have affected our semantic representations much. First, several previous studies on word embeddings for machine translation showed similarity in the semantic representation across various languages^41,42^. Second, English and Dutch are both Germanic languages and share additional similarities in terms of their phonetical, lexical and grammatical structure. Finally, because much of the semantic content in this study is based on the visual stream of information, we expect it to generalize well across languages, and better compared to, for example, non-sensory, more abstract type of information.

### Capturing of fundamental semantic distinctions in the extracted semantic components

Whether it would make sense to seek a relationship between the extracted semantic components and the brain responses depended of the capability of the taken approach to capture meaningful distinctions and elements of semantics that drove the difference between various fragments of the film. We were particularly interested in exploring the space of the extracted semantic components and determining whether they captured interpretable semantic information.

We found that the first five principal semantic components explained ~ 70% of all variance, suggesting that most of the semantic variability was captured by five fundamental semantic components. Each of the remaining components accounted for less than 5% of variance in the data and most of them (34 out of 45) for less than 1%.

**Figure 2:**
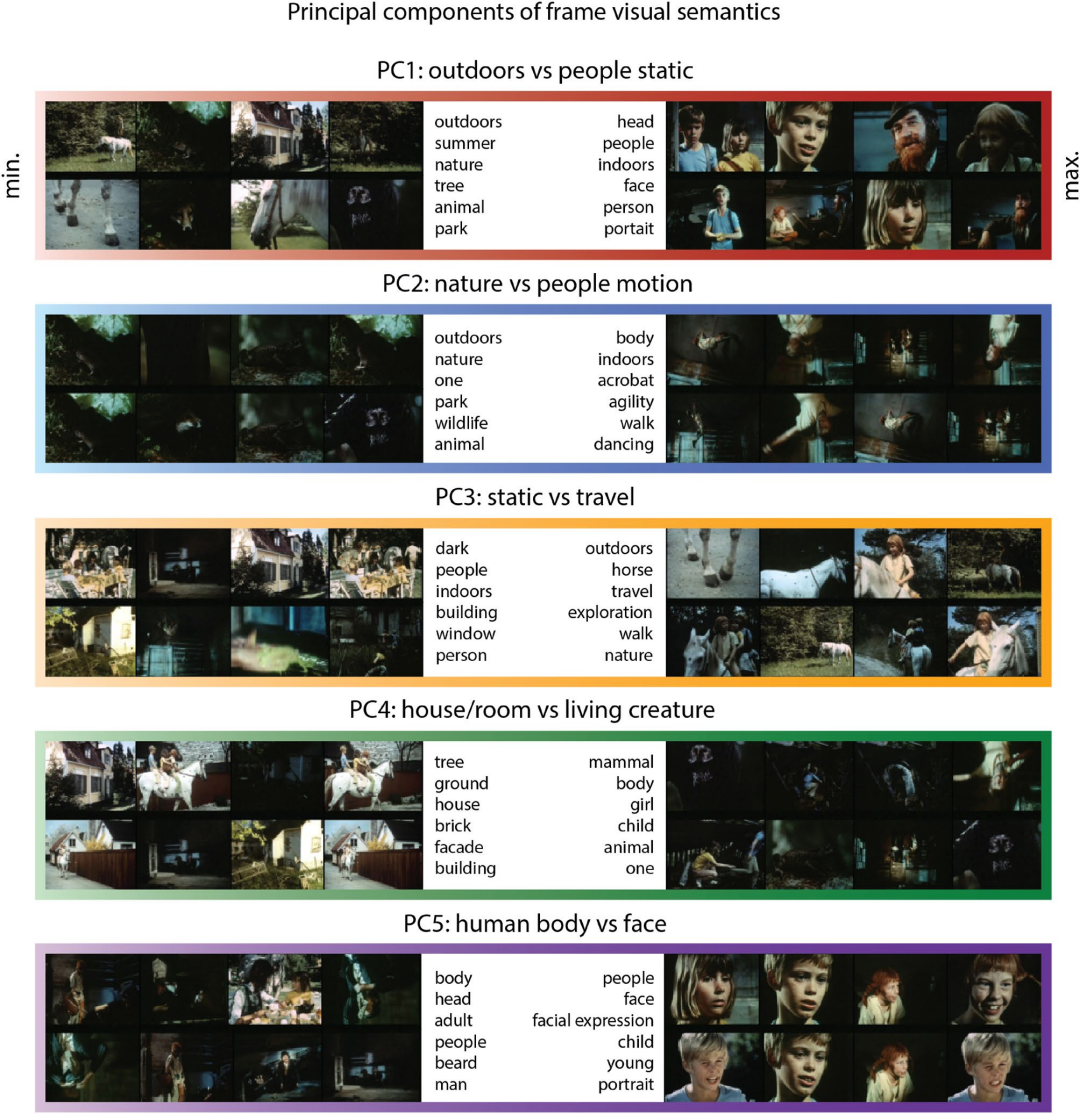
Interpretation of the top five semantic components in the film’s visual stream. For each component we selected frames with bottom 10% (negative) and top 10% (positive) values. Thus, these selected frames express the range of semantic information captured in each component. The colors are unique identifiers of each components that are also used in later figures. The words associated with either bottom 10% or top 10% of frames per component are the most frequent concept labels present in these selected frames.

To examine the top five extracted semantic components, we ranked the frames with respect to their values along each semantic component. For each component we selected frames corresponding to the bottom and top 10% of values and visualized them (Fig. 2). In addition, we computed histograms of concept labels associated with the selected frames. Thus, the bottom 10% of frames together with their label histograms exhibited low values along a specific component, and the top 10% exhibited high values.

We observed that the first semantic component (34% explained variance) represented presence or absence of people in the frame. The second component (17% explained variance) differentiated between human movement and non-human movement or general nature scenes. The third component (11% explained variance) differentiated between scenes with movement (including travel and walking) and static scenes. The fourth component (9% explained variance) reflected differences between scenes with landscapes (including houses, rooms and other spaces) and portrait-like scenes (person or animal). Finally, the fifth semantic component (5% explained variance) captured human faces and differentiated them from scenes with human bodies and frames without people.

To assess the relationship between the semantic components and the labels we performed post-hoc statistical testing by fitting a linear regression to predict the values along the five semantic components using the concept labels per frame. The fit was significant for each of the top five semantic components (${R}^{2}>0.99, F\left(128; 9,675\right)=14,490, p\ll 0.001$) and yielded regression weights for each of the components. The highest and lowest weights corresponded well to the histograms of concept labels for the top and bottom 10%, respectively, in that the highest weights for the first semantic component were assigned to labels ‘people’, ‘man’, ‘girl’, ‘adult’ and the lowest weights—to ‘park’, ‘tree’, ‘animal’, ‘outdoors’. The highest weights for the second component were assigned to labels ‘climb, ‘acrobatics’, ‘music’, ‘agility’ and the lowest weights—to ‘wildlife’, ‘outdoors’, ‘people’, ‘dark’, etc. Comprehensive lists of highest and lowest weights as well as the 2D visualization of the overall structure of the semantic components (based on a t-SNE projection, see Methods for details) are shown in Supplementary Material (Figures S1, S2).

### High accuracy of predicting the neural responses based on the extracted semantic components

**Figure 3:**
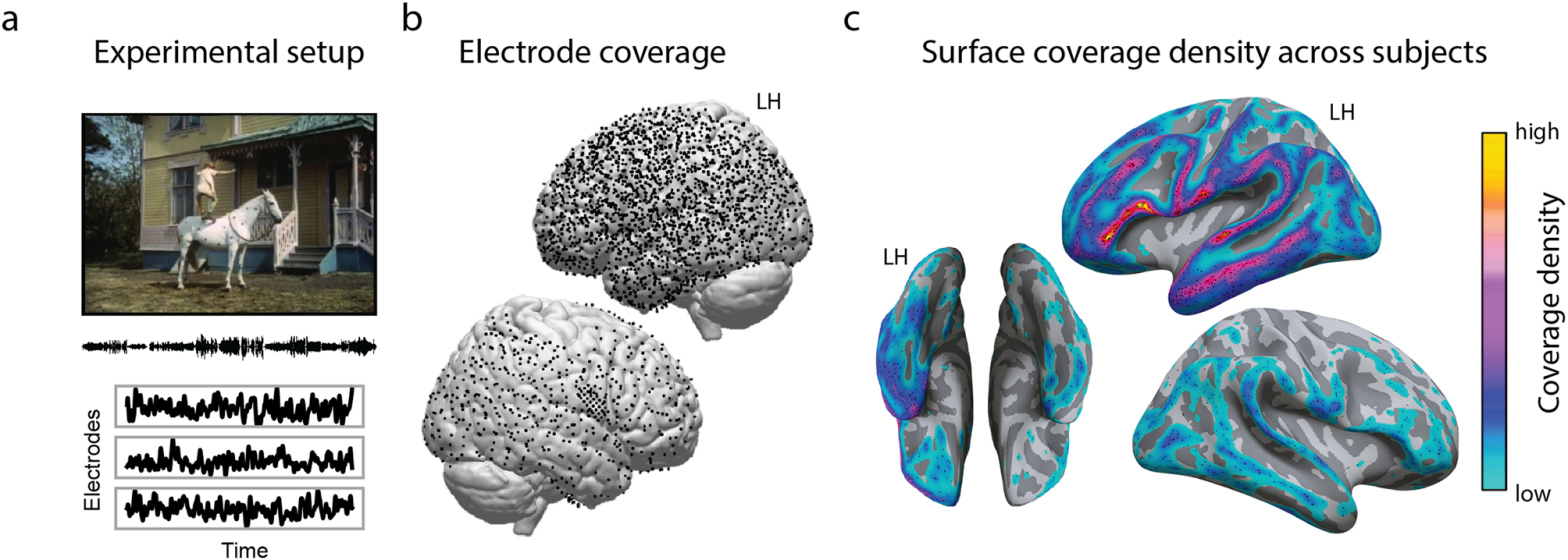
Overview of the ECoG film-watching experiment. (**a**) Experimental setup. The audiovisual film was presented to each participant while their neural responses were collected with ECoG electrodes. (**b**) Total electrode coverage over 37 participants. The panel shows a clear bias toward covering the left hemisphere. Each single subject electrode location is projected onto the MNI common brain. (**c**) The surface representation of the coverage density. Each single subject electrode location was assigned a value of one and projected to a regular grid in the average Freesurfer space. The resulting overlay shows the density of coverage over all individual participants. The panel shows the same bias for the left hemisphere as well as better overall coverage of inferior frontal and temporal cortices. Considerable coverage of inferior temporal and fusiform regions can be seen, whereas calcarine sulcus and lingual gyrus are covered quite scarcely. See “Methods” for more details about the projection to a uniform regular grid on the average surface.

To assess whether the five semantic components mapped onto the neural responses to the film (Fig. 3), we fitted a neural encoding model^42–45^ where high frequency band (HFB) neural responses across all electrodes were predicted based on the extracted semantic components. Previous studies have shown that the HFB amplitude closely correlates with neuronal firing rates on the neocortex and with fMRI blood-oxygenation-level-dependent response (BOLD)^46–49^.

To account for the complex multimodal nature of the film prior to fitting a model on the extracted semantic components, we first regressed out parts of the neural signal associated with the auditory stream of the film (SI Figure S3). A ridge linear regression was then applied on the residual neural data to predict whole-brain HFB ECoG responses to the film using the semantic components, with Pearson correlation between predicted and observed HFB responses as the performance metric. The reported performance was calculated in the held-out test set and was cross-validated (see “Methods” for details).

**Figure 4:**
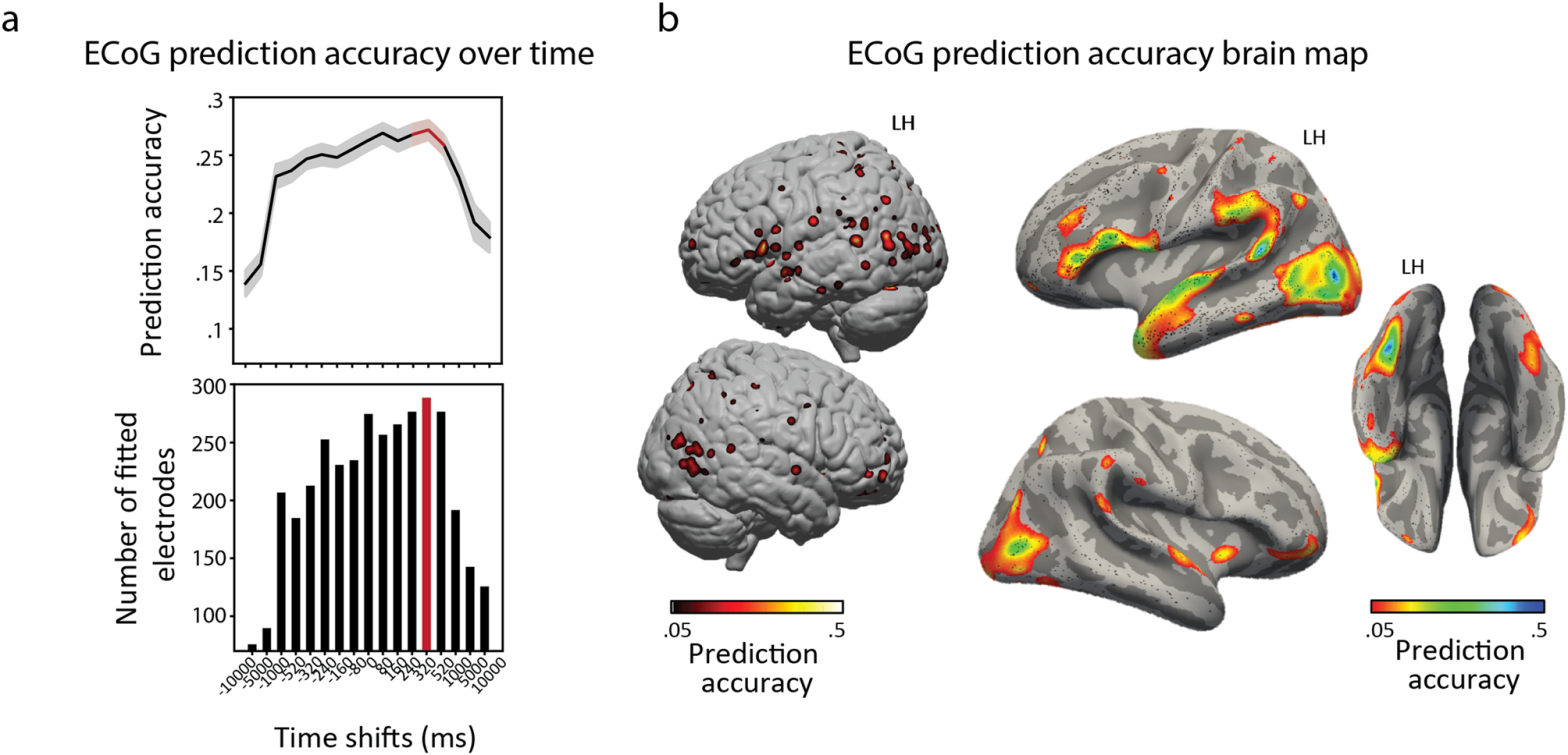
Fitting the neural responses based on the extracted semantic components. (**a**) Prediction accuracy (measured as Pearson correlation between predicted and observed HFB responses) for various time shifts of the semantic components. Prediction accuracy was cross-validated over five folds. Testing different time shifts was done to account for a delay in neural processing of the high-level visual information. Top panel shows the mean prediction accuracy for all significant electrodes ($p<1\times {10}^{-5}$, Bonferroni corrected for the total number of electrodes) per time shift. Shading represents standard error of the mean. Bottom panel shows the number of electrodes with a significant fit per time shift. The red bar indicates the model with top average prediction accuracy as well as highest number of electrodes with a significant fit, which corresponds to a time shift of 320 ms. (**b**) Cortical map of the prediction accuracy for the time shift of 320 ms ($p<1\times {10}^{-5}$, Bonferroni corrected for the total number of electrodes). Left panel shows a volume-based plot. Each cross-validated prediction accuracy value was assigned to the center coordinate of the corresponding electrode and projected to the MNI common space. Individual electrode locations were normalized to the MNI space using subject-specific affine transformation matrices obtained with SPM8. For the visualization purposes a 2D Gaussian kernel (FWHM = 8 mm) was applied to the coordinate on the MNI brain volume corresponding to the center of the electrode, so that the projected values (e.g. prediction accuracy) faded out from the center of the electrode toward its borders. Right panel shows a surface-based plot of the cross-validated prediction accuracy at the time shift of 320 ms. It shows the same prediction accuracy values as the left panel but projected on the surface for a better display of the fit in lateroocipital and fusiform cortices. See “Methods” for more details about the projection to a uniform regular grid on the average surface.

Given that semantic processing is a high-level cognitive process that has been shown to require a substantial delay relative to the stimulus onset, we also tested which time shift relative to the stimulus onset was best for predicting the neural responses (Fig. 4a). A separate ridge linear regression model was fitted at every time shift within the range of – 10 to 10 s. The highest accuracy of prediction was found at a lag of 320 ms after the stimulus onset. Even though longer lags displayed progressively lower prediction accuracy, multiple lags around the stimulus onset led to a high accuracy of the fit. This was most likely due to the high autocorrelation of the semantic data (SI Figure S4).

Considering the cortical map of the prediction accuracy, the best accuracy was achieved for occipitotemporal, parietal and inferior frontal cortices (Fig. 4b). The prediction accuracy ranged from $r=0.11$ to $r=0.52$ (cross-validated Pearson correlation between predicted and observed HFB responses, 453 electrodes, 16% of all electrodes) at $p<0.001$, Bonferroni corrected for the number of electrodes.

### Highly specialized cortical networks triggered by individual semantic components

Given that the prediction accuracy was high in a number of brain regions bilaterally, we wondered whether all of the observed regions were involved in semantic visual processing to the same extent or whether there was a degree of specialization for some brain regions in responding to specific semantic components.

**Figure 5:**
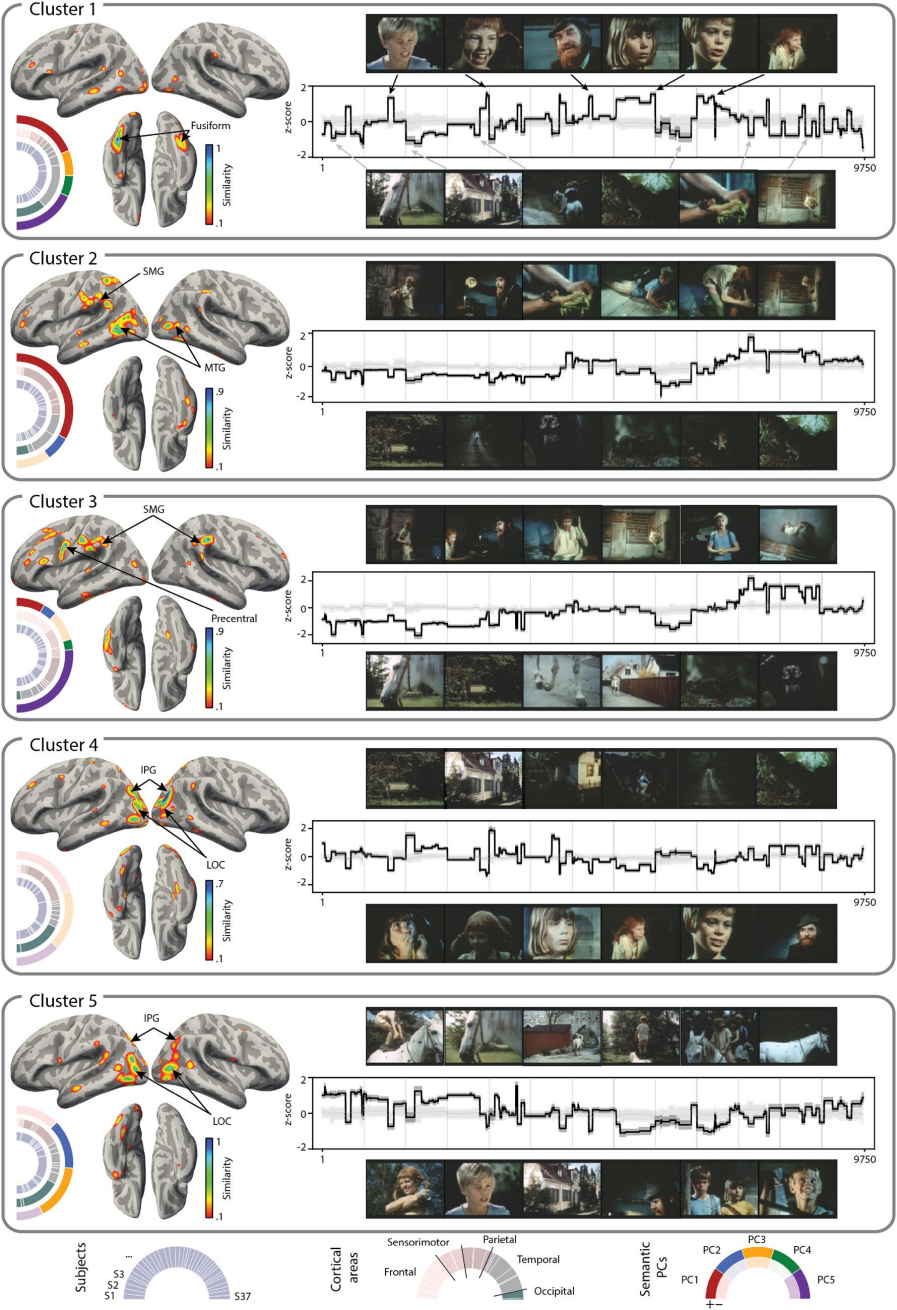
Distributed functional cortical networks associated with each individual semantic component. Each cluster’s profile contains diverse information including the cortical distribution of the electrodes contributing to that cluster (1), distribution over single subjects to show that all reported clusters included multiple subjects (2), distribution over cortical areas (3), distribution over the five semantic components (3), activation time course of the cluster (4) and frames that trigger and deactivate the cluster (5). Cortical maps are the surface-based projections of the similarity of each electrode in the cluster to the cluster exemplar. The similarity is measured as Pearson correlation of the $\beta$-weights across all electrodes. The $\beta$-weights are vectors of regression coefficients over 50 semantic components produced by the ridge linear regression fit. Distributions over subjects (inner pie charts), per cluster, show a ratio of all electrodes that came from each single subject to the total number of electrodes. None of the reported clusters were subject-specific, meaning that no more that 30% of electrodes per cluster came from a single subject. Distributions over cortical labels (middle pie charts) are color-coded and only five groups of labels are highlighted: frontal, sensorimotor, parietal, temporal and occipital regions. The proportion of labels within each color-coded region is also informative, such that in Cluster 1, for example, the largest contributing label is the fusiform gyrus, which is a temporal region. It is not additionally color-coded, but one can see that among the temporal regions there was only one with the largest contribution (which corresponds to the fusiform gyrus). Other times, the contribution can be more equally distributed over multiple regions with the same color-coded labels. Distribution over the semantic components (outer pie charts) was calculated by performing a signed difference test on the top 10% of frames associated with peaks in the cluster’s activation time course and the bottom 10% of frames associated with dips in the cluster’s activation time course. Per cluster, the signed difference test (two-sided Wilcoxon ranked test) was performed by comparing values along each semantic component between ‘peak frames’ and ‘dip frames’. The pie charts represent the test statistic, significant at $p<0.001$ (Bonferroni corrected for the number of clusters and semantic components), adjusted for the decreasing percentage of explained variance from the first to the fifth semantic component (see “Methods” for details). The cluster activation time courses show the dot product of the semantic components and the $\beta$-weights of the exemplar of each cluster. Shading represents the standard error of the mean calculated on the dot product using $\beta$-weights of all electrodes of the cluster. Examples of frames associated with peaks in the cluster’s activation are displayed above the activation time course. Examples of frames associated with dips in the cluster’s activation are shown below the activation time course.

Investigation of the $\beta$-weights of the linear encoding model at the optimal time shift of 320 ms showed that distinct cortical networks were engaged in processing of the visual semantics of the film. Specifically, in order to identify groups of electrodes with similar $\beta$-weights across 50 components, and thus similar semantic encoding profiles, we applied a clustering approach to all $\beta$-weights[32,38]. As a result, we identified a number of clusters, each characterized by a distinct cortical network as well as a ratio of contribution of the top five semantic components to the activation of that cluster (Fig. 5). Thus, we observed for instance, that cluster 1 comprised electrodes in the lateral fusiform gyrus, and the semantic components encoding human presence (first semantic component) and human faces (fifth semantic component) contributed most to its activation time course.

Several cortical networks (clusters 2, 3 and 5) responded to the semantic components capturing motion. These networks comprised electrodes in supramarginal, precentral and posterior middle temporal gyri. The cortical network in cluster 2 seemed to be more specific to hand movement and object interaction (second semantic component), whereas the cortical network in cluster 3 seemed be more related to encoding of facial movement and facial expressions (combination of first, second and fifth semantic components). Cluster 5 appeared to be less sensitive to human presence in the frames but responded to biological motion and the semantic component of travel and transportation (third semantic component).

Cluster 4 was associated with presence of landscapes and static shots. Biological movement seemed to cause dips in its activation time course. Both clusters that included lateral occipital and dorsal parietal regions (clusters 4 and 5), favored frames without people presence and were deactivated by frames with human faces.

Not all of the cortical networks uncovered through clustering were as easily interpretable through the analysis of their distribution over the top five semantic components. Remaining clusters with contribution from electrodes of many subjects still exhibited high anatomical consistency and included electrodes in inferior frontal gyrus (mostly pars opercularis) as well as posterior and anterior sites along the superior temporal gyrus (SI Figure S5). It is unlikely that these cortical networks can be connected to tracking of the auditory information due to our control for interaction between auditory and visual streams of the film. In addition, the activation time courses of these clusters do not seem to follow the block design, nor do they exhibit significantly larger correlation with the audio envelope compared to other clusters.

### Control for the low-level visual features

Because the semantic components we used here were based on the output on the visual concept recognition model trained on raw image data, we aimed to implement some form of verification that the reported results were indeed due to the semantic processing rather than processing of lower-level visual features. First, we were able to confirm that pixel-level and Gabor-based visual features were dissociated from the extracted semantic components (two-sided Wilcoxon signed-rank tests: $Z=-3,118, p\ll 0.001$ for pixel values and $Z=-5,928, p\ll 0.001$ for Gabor features). Next, we used low-level visual features to predict the neural responses to the film with an intention to compare its prediction accuracy to the accuracy of the model that used the semantic components at the temporal shift of 320 ms. Overall, we observed that the fit based on low-level features was considerably poorer, with prediction accuracy ranging from ${r}_{pix}=0.11$ to ${r}_{pix}=0.45$ (6% of all electrodes) for colored pixel values and ${r}_{gab}=0.11$ to ${r}_{gab}=0.41$ (7% of all electrodes) for Gabor features. The overall difference in the whole-brain prediction accuracy was statistically significant: ${Z}_{sem-pix}=16.13, p\ll 0.001$ and ${Z}_{sem-gab}=15.27, p\ll 0.001$, Bonferroni corrected for the number of electrodes, as assessed with a one-sided Wilcoxon signed rank test. All electrodes with a significant fit with either model ($sem$ or $pix$ and $sem$ or $gab$) were used in the comparison. The medians of the prediction accuracy per model over all electrodes used in each comparison are displayed in SI Figure S6.

It is important to note that here we only had limited ECoG coverage in early visual cortex (Fig. 3c). Apart from that, the film watching experiment did not have a fixation point in the center of the screen. Thus, unsurprisingly, using low-level visual features to predict the neural responses provided a significant fit in a limited number of electrodes outside of the early visual cortex. Overall, the brain map for the difference in the fit (${r}_{sem}-{r}_{pix}$ and ${r}_{sem}-{r}_{gab}$, SI Figure S6) showed a similar cortical distribution as the map for the fit using the semantic components (Fig. 4b) confirming that the low-level visual features did not affect the results of the semantic encoding.

### Emergence of visual semantics from low-level visual features

**Figure 6:**
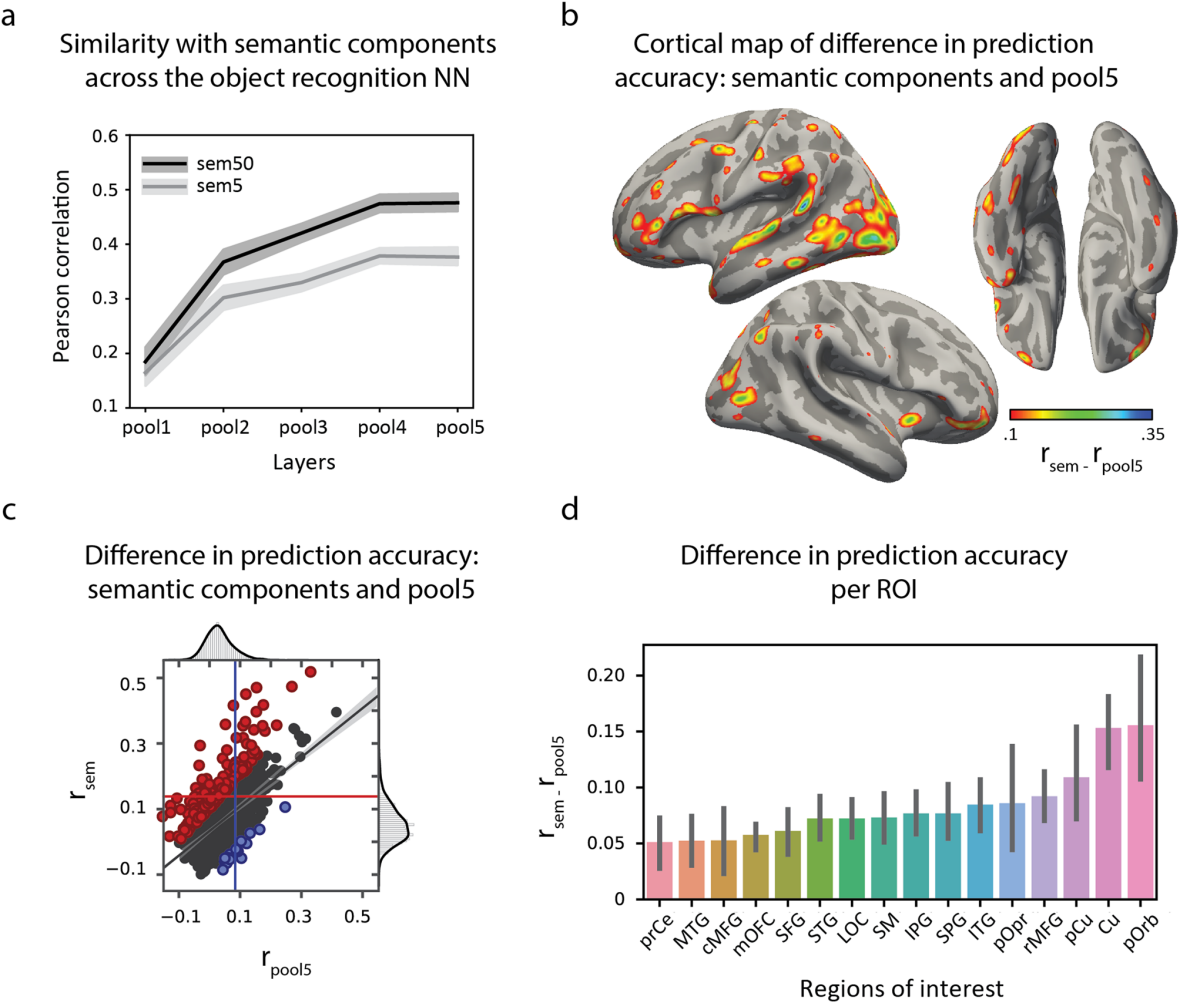
Gradual emergence of the semantic information from low-level visual features. (**a**) Similarity of frame representations between each intermediate layer of the automatic visual object recognition model and the semantic components used to fit the brain data. We used all so-called *pooling* layers of the object recognition model as intermediate layers. First layer (*pool1*) is placed quite early in the model (it is preceded by only two convolutional layers), whereas the last layer (*pool5*) is located very deep in the model (followed by two dense layers and a probability output layer). The similarity with the semantic components is measured as Pearson correlation of all pairwise frame comparisons between each pooling layer and the semantic components (see “Methods” for details). Dark grey line shows similarity between each pooling layer and all 50 semantic components, light grey line shows similarity between each pooling layer and only top five semantic components. In both cases the shading represents 95th confidence interval based on the bootstrapping procedure (sampling of 1,000 frames 10,000 times). (**b**) Cortical map of the difference in prediction accuracy between the fit using features of the last pooling layer (*pool5*) and the semantic components. (**c**) Scatter plot showing the difference in prediction accuracy between the brain fit using *pool5* (${r}_{pool5}$) and the semantic components (${r}_{sem}$). The results are shown for the models fitted at a 320 ms temporal shift of the brain data with respect to the stimulus onset. Each point represents a cross-validated accuracy per individual electrode. Red-colored points denote electrodes with ${r}_{sem}-{r}_{pool5}>0.1$, blue-colored points denote electrodes with ${r}_{pool5}-{r}_{sem}>0.1$. Red-colored line shows median prediction accuracy over all electrodes with a significant fit for the semantic components, blue-colored line shows the same value for *pool5*. (**d**) Significant difference in the prediction accuracy for individual regions of interest (ROI). Each bar shows a difference in median prediction accuracy values (${r}_{sem}-{r}_{pool5}$) per ROI. Error bars represent 95th confidence interval based on the bootstrapping procedure (resampling of the electrode accuracy values per ROI 10,000 times). We only show the ROIs, for which the difference in the median prediction accuracy (${r}_{sem}-{r}_{pool5}$) was above zero (based on the confidence intervals of the bootstrapping distributions): pOrb (pars orbitalis), Cu (cuneus), pCu (precuneus), rMFG (rostral middle frontal gyrus), pOpr (pars opercularis), ITG (inferior temporal gyrus), SPG (superior parietal gyrus), IPG (inferior parietal gyrus), SM (supramarginal gyrus), LOC (lateral occipital complex), STG (superior temporal gyrus), SFG (superior frontal gyrus), mOFC (medial orbitofrontal cortex), cMFG (caudal middle frontal gyrus), MTG (middle temporal gyrus), prCe (precentral gyrus). All ROIs defined in the Desikan–Killiany atlas (34 regions in total) were used in this analysis.

Thus, we have shown that the observed prediction accuracy for the whole-brain responses was not due to low-level visual features. Since the visual concept recognition model was trained to extract high-level semantic information (concept labels) from raw image data, somewhere along the layers of this deep artificial neural network, higher-level semantic visual features were bound to emerge. We were curious whether we would be able to track the gradual buildup of these semantic representations throughout the visual concept recognition model and whether this buildup would be supported by the neural data. To this end, we investigated the relationship between the semantic components and each intermediate layer of a publicly available object recognition model called VGG16^50^, similarly trained to recognize objects in images by passing them through a set of convolutional layers. For simplicity, we only considered the *pooling* layers of the VGG16 model (see “Methods” for details). We found a gradual increase in similarity between frame representations from the first to the last intermediate layer of the object recognition model with the semantic components (Fig. 6a). Interestingly, the fit to neural data based on each individual intermediate layer of the object recognition model also showed a gradual increase in prediction accuracy (from the first to the last layer) together with the spread of the location of the best fitted electrodes from occipital toward temporal and parietal cortices (SI Figure S7). In particular, we observed that the activity in the fusiform gyrus was fitted better with later layers of the VGG16 (*pool4* and *pool5*), eventually showing little difference with the fit using the semantic components compared to a much larger difference at the earlier layers (*pool1* and *pool2*, SI Figure S7).

We then used the last intermediate pooling layer of the object recognition network (layer *pool5*, which we would expect is sensitive to complex patterns of object parts and general object shapes based on the existing work^25,51^) and compared the neural fit on *pool5* features to the fit on the semantic components, both estimated at same temporal shift of 320 ms. The prediction accuracy ranged from ${r}_{pool5}=0.11$ to ${r}_{pool5}=0.41$ (6% of all electrodes) for the fit using *pool5* features. All electrodes with a significant fit with either model ($sem$ or $pool5$) were used for the comparison of the models. The difference in prediction accuracy between the fit on the semantic components and the fit on *pool5* features was observed for 28% of all electrodes favoring the fit on the semantic components (${r}_{sem}-{r}_{pool5}>0.1$, Fig. 6b). The overall difference in the whole-brain prediction accuracy was statistically significant: $Z=16.51, p\ll 0.001$, Bonferroni corrected for the number of electrodes, as assessed with a one-sided Wilcoxon signed rank test (Fig. 6c). The medians of the prediction accuracy per model over all electrodes used in each comparison are displayed in Fig. 6c. Electrodes in the occipitotemporal, parietal and frontal cortices showed a better fit using the semantic components compared to the fit on *pool5* features (Fig. 6d).

Overall, these results indicated a gradual emergence of the semantic features from the low-level visual information in the object recognition model. At the same time, even the top layer of the object recognition model (*pool5*) provided an inferior fit of the neural data compared to the semantic components. The cortical areas with the overall significant difference in the prediction accuracy were high-level cognitive processing regions including various subareas of the frontal cortex (superior, middle, inferior frontal gyri and medial orbitofrontal cortex), superior and inferior temporal, parietal and motor regions. Of the occipital regions, only cuneus and precuneus showed a significant difference.

### Contribution of the language model to the extracted semantic components

Finally, having seen that the semantic components provided a better fit for the brain responses throughout high-level cognitive areas compared to the top pooling layer of the visual neural network, we wondered whether the language model contributed to the model accuracy.

To address this point, we fitted another ridge linear regression model using binary vectors of concept labels to predict HFB neural responses. We then compared the prediction accuracy with the model that used the semantic components, both estimated at same temporal shift of 320 ms. The prediction accuracy ranged from ${r}_{labels}=0.11$ to ${r}_{labels}=0.51$ for the fit using the binary labels. We found that even though there did not seem to be a specific brain region where the accuracy was significantly better for the semantic component model, the model that used the semantic components on average provided a better prediction accuracy across the cortex (one-sided Wilcoxon signed rank test: ${Z}_{sem-labels}=11.82, p<0.001$, Bonferroni corrected for the number of electrodes with a significant model fit). This difference was significant even though the difference in median accuracy values was not large: $median({r}_{sem})=0.14$ and $median({r}_{labels})=0.12$. Importantly, more electrodes were fitted significantly well using the semantic components compared to the binary concept labels: 453 electrodes and 338 electrodes, respectively (at $p<0.001$, Bonferroni corrected for the total number of electrodes).

## Discussion

In the present study we show that the automatically derived semantic properties of the visual narrative in a short film are captured in distributed cortical networks, each associated with distinct semantic components. In particular, we were able to combine recent advances in both visual object recognition and natural language processing to develop a semi-automatic approach to extract semantic components from the visual stream of the film. Modelling the associated neural responses on the basis of the semantic components resulted in significant prediction accuracy peaking at 320 ms after the frame onset, primarily in occipitotemporal, parietal and inferior frontal cortices. Investigation of the model weights showed that distinct cortical networks were engaged in processing of the visual semantics of the film with lateral fusiform gyrus processing faces, supramarginal, motor and posterior middle temporal regions processing movement, and lateral occipital regions processing complex static scene information.

### Fitting neural responses with semantic information derived through automatic processing of stimuli

Previous research on neural encoding models using deep neural network representations shows their potential in explaining brain activity. Multiple studies have reported that activation in both early and secondary visual cortex reflects similar representations of visual stimuli as the trained artificial neural networks^24,25,28^. High-level semantic distinction and object representation have been traditionally associated with inferior temporal and fusiform cortices^6,7,9,52^, and it has been shown that activation patterns in these regions exhibit similarity with representations learned by top layers of object recognition neural networks^34,53^.

Language models that extract semantic properties of words by learning their co-occurrence patterns have also been successfully related to neural data through either encoding^36^ or decoding^54,55^ approaches. Exploiting the co-occurrence patterns to study encoding of meaning in the brain has proven fruitful regardless of whether the co-occurrence patterns were extracted through an artificial neural network^36,37,55^ or simpler corpus-based approaches^35,56^, underlining the importance of contextual information in semantic representation.

Here, an important step forward from this impressive previous work is the combination of the representations from different domains for predicting brain responses. We combine advances in both visual object processing and natural language processing to obtain rich semantic representations of the film’s visual stream that are informed by a language model. We find that this combined approach provides a better fit for whole-brain neural responses to a complex audio-visual narrative, than concept labels alone, even after having manually corrected their assignment.

It remains an open question whether the concept labels and semantic representations used in this study are the optimal way to represent meaning in the visual stream of the film. What it means to be ‘optimal’ should also be clearly defined, as one may consider optimal the semantic labeling that best fits the content of each individual frame. Alternatively, the semantic labeling can be considered optimal if it best describes the storyline of the video or recognizes the content of the situation displayed (for example, who is doing what to whom)^57^. It can also be argued that a linguistic caption, which can also be automatically inferred from the image data^58,59^, is a better way to annotate an image compared to a set of individual labels. Ultimately, as neuroscientists we are interested in the semantic labels or representations that above all explain the neural responses in high-level associative cortices. Bearing this in mind, in this study, we chose a fairly simple and straightforward model that combined both perceptual and linguistic information for extraction of the semantic representations. At the same time, many other frameworks in computer vision (^57,60–62^ among others) could be used as a basis for neural encoding models, and we do not have the evidence to claim that the approach presented here is superior. A comprehensive comparison of various image labeling methodologies is beyond the scope of this study but warrants further investigation.

### Gradual emergence of the semantic representations from low-level visual input

One of the main reasons why artificial neural network models are so interesting from the neuroscience point of view is their ability to extract hierarchical representations capturing transitions from low-level raw input data, such as images, to high-level semantic distinctions.

Previous research in visual neuroscience used data from fMRI, magnetoencephalography (MEG) and ECoG to show that increasingly complex representations learned by an image-based deep learning model, translated into a gradient along the cortical areas involved in visual object recognition. Along this gradient, responses in early visual cortex were fitted best by representations in early layers of the deep learning model, whereas responses in fusiform and inferior temporal cortex were fitted best by representations in later, more object- and category-specific layers of the deep learning model^24,27,34,53^.

The present study corroborates the evidence that high-level semantic representations emerge gradually throughout the deep artificial neural network, and that this gradual shift maps onto neural data as well. That is, larger similarity of the later layers of the visual object recognition model with the semantic components was reflected in a better fit to the brain responses in high-level areas including occipitotemporal, parietal and frontal regions. Our approach seems to provide a better fit compared to the top layer of the visual object recognition model in many high-level cognitive areas. This once again underlines the contribution of the use of manually corrected labels combined with a language model that captures semantic distinctions between the labels.

### Distributed functional cortical networks each encoding an individual semantic component

An attempt at combining the visual concept recognition model with a context-based language model has previously been made using fMRI^36^. However, one of the strengths of the present study is the focus on exploring the semantic space of the extracted high-level representations, and encoding of individual semantic components in the neural responses. Thus, we show that the semantic distinctions observed along each of the top principal semantic components are associated with activation of specific functional cortical networks. The ability to uncover this mapping and interpret the cortical activity through processing of variance along distinct continuous semantic dimensions is the main contribution of this work.

The adopted approach separated the functional cortical networks associated with visual perception of human movement, human faces, general movement, landscapes and static scenes. The involvement of the lateral fusiform gyrus in processing of human faces has been reported in a large body of previous research^6,9^, focusing on its role in identity perception^63,64^. Interestingly, here the human faces seemed to be part of a semantic dimension, with nature, landscape and movement frames on one end and human faces on the other. This finding resonates with theories on existence of a so-called animacy continuum that drives difference in the neural responses to various visual stimuli^39,65^.

Processing of human movement in our study shows involvement of sensorimotor cortex, supramarginal gyrus and middle temporal region, or MT. The MT region is the classical area reported in perception of motion in general, whether it is movement of humans, objects or dot patterns^11,66,67^. However, the other two regions are reportedly involved in a more abstract level of motion perception. Sensorimotor cortex (pre- and postcentral gyri) has been implicated in visual perception of human action^68,69^. Supramarginal gyrus with an extension to anterior intraparietal sulcus has been reported to be involved in perception of spatial relations as well as complex human and animal body movements^70,71^, and of hand movement and interaction^67,72^.

Another functional cortical network that emerged from this analysis is a combination of the lateral occipital cortex with inferior and superior parietal gyri. It is associated with processing of scenes, places, salient regions and possibly of multiple objects in general^73–75^. Involvement of the parietal regions indicates simultaneous encoding of the spatial relations of the objects within a scene^5,76^. In the present work, coordination of this network with the MT region occurs during perception of the movement in a scene or through a specific place, consistent with the view on integrative (form-motion) function of the lateral occipital-temporal cortex^77^.

### Using continuous semantic components to map the patterns of neural activity

The holistic approach in the current study complements what has been learned from research using traditional approaches to studying visual semantics and object categorization in controlled^63,78,79^ and naturalistic^80–82^ experimental paradigms using a variety of the neural recording techniques (fMRI, MEG, ECoG, stimulation techniques). But, rather than addressing hypotheses by formulating specific questions and constraining the experimental paradigm, the current bottom-up analysis of data obtained in a natural context maps deep structures of visual input onto neural activity. Instead of looking to decode pre-selected discrete semantic categories we focused on the variance along individual orthogonal semantic axes^37,39,83^ extracted from the raw input itself. The fact that the categories that have traditionally been investigated, such as faces, places, movement and body parts turned out to be the principal components in the visual domain of the feature film, underscores the usefulness of a more holistic approach for investigating neural substrates of attribution of semantic meaning to visual input. As such, the presented findings constitute an indication of the largest contrasts of semantic features that constitute a key dimension in the visual input that cortical networks respond to, which may shed some light on how the brain attributes semantic meaning in a natural situation.

### Using ECoG for studying semantic representations in the human brain

Notably, the present study is among the few that investigate semantic processing in ECoG neural responses^84–88^. The majority of the work we have previously referred to when interpreting our results use fMRI and MEG for studying semantic representations in the brain.^24,26,78^ While combining the two allows for compensating for each modality’s individual drawbacks, neither technique samples the brain responses directly from the neural tissue. Intracranial neural recordings, such as ECoG, do not only offer both high temporal and high spatial precision of the signal, they are also characterized by exceptional signal-to-noise ratio. The latter allows for analysis of the HFB component of the neural signal that is often linked to the local spiking rates and is associated with bottom-up local information processing.

Despite its numerous advantages, ECoG data are rare. Most ECoG studies report only a limited number of subjects (typically below ten), and therefore have rather limited brain coverage that makes it difficult to investigate whole-brain responses to semantic content^84–88^. Here, we were able to overcome this limitation as we were able to collect ECoG data from a large number of participants (37 subjects), which allowed us to investigate HFB activity during semantic processing across a large number of brain regions during naturalistic audiovisual stimulation. Importantly, despite the high temporal autocorrelation of the semantic data, we were able to show a time-locked response to the semantic content in HFB signal. We also observed specialization of the different cortical networks in processing specific semantic information. It is difficult to predict how these results would compare to analogous recordings with MEG, for example. The current results make a strong case for a possibility of studying whole-brain responses to complex naturalistic stimuli by effective processing of a large ECoG dataset and the corresponding stimulus data.

### Limitations and further directions

The present study has a number of limitations. For instance, our interpretation of the cortical patterns supporting semantic processing is limited by the ECoG coverage. Not all cortical regions were sampled uniformly by ECoG electrodes and activity in deep and folded regions was not recorded. Yet, we do not claim to explain full-brain neural activity underlying semantic processing, but rather reveal that high-level properties of the visual stream of the film explain part of the variation of cerebral responses in distributed cortical networks. Despite the limitation in coverage, the number of ECoG participants (37 subjects) is relatively large for an ECoG study and the reported results are likely to generalize across individuals.

Given the high correspondence of the HFB signal to both the local neural spiking activity^46,49^, and the BOLD signal^47,48^, combined with the ability of ECoG to capture crisp HFB activity, the present work only focused on that component of the neural response. A promising extension of this work would involve focusing on the cross-frequency coupling during semantic processing of the visual narrative.

Another limitation lies in the experimental material. Being part of the battery of standard clinical tasks developed specifically for diagnostic purposes, the experiment did not use material of a full feature film, but comprised a reduced subset of 30 s excerpts of a feature film (in total, 6.5 min long), edited together for a coherent story. Such a stimulus accommodated the main purpose of the clinical task to compare the speech and music sound blocks of the film (30 s each). We minimized the effects of this stimulus manipulation by regressing out the block structure and the auditory envelope from the cortical responses. Nonetheless, this manipulation might have still affected our data and overall, the task structure resulted in a limited amount of data compared to using a full feature film material.

We also observed that the visual stream of the film was characterized by high consistency of semantic information in consecutive frames. Logically, this makes sense as in a real world the information we perceive shares a lot of high-level abstract properties over consecutive time points resulting in high autocorrelation of the semantic properties of perceived input. Even though it appears to be an inherent feature of naturalistic semantic processing, it made the investigation of the dynamics of semantic processing rather limited.

Finally, the contents of any feature film in general contain material that people like to observe, and logically are likely to include people, actions, movement, various locations and so on. This could lead to an inherent bias in the type of semantic information that can be extracted from such material. It could also explain the correspondence of the semantic components extracted here with features investigated in isolation with traditional approaches (faces, movement, places, etc.). More research with more extended stimulus material is needed to estimate the bias and its effect on our understanding about the way semantic information is represented in the human brain.

In addition, we would like to note that our three-stage semi-automatic approach for extraction of semantic information in the visual stream of the film is only one of the many possible pipelines that make use of automatic processing of perceptual and language information for extraction of semantic meaning. Similarly, we do not claim that this approach is optimal for semantic labeling of image or film frames. As our main goal was to extract meaningful semantic representations and search for their encoding in the neural responses, we performed no comparisons with alternative labeling methodologies (^57,60–62^ among many others). An interesting extension of this work could focus on further methodological developments and comparisons with alternative frameworks that use more sophisticated language models^19^, combine linguistic and perceptual information differently if at all, or show other ways to estimate the dimensionality of the semantic data. Finally, more complex approaches for modeling semantics of the feature film can be created by combining information from both the auditory and the visual channels of information.

### Conclusions

In the present study, we combined advances in computer vision and natural language processing to automatically extract complex semantic information from the visual stream of a short feature film. When fitted to predict whole-brain neural responses, these continuous semantic features triggered activation of distinct functional cortical networks, each associated with an individual semantic component of the visual narrative. These results underscore the potential of computational models that extract high-level semantic information from input data to offer insight about how the human brain processes visual information and forms semantic representations of the perceived world.

## Methods

### Film stimulus

For the film-watching experiment we used a 6.5 min short movie, made of fragments from “Pippi Langkous” (Pippi Långstrump, 1969) edited together to form a coherent plot. The task was part of the standard battery of clinical tasks performed with a purpose of presurgical functional language mapping. Therefore, here we worked with a dataset originally collected for diagnostic purposes using restricted experimental material. The film consisted of 13 interleaved blocks of speech and music, 30 s each (seven blocks of music, six blocks of speech). The movie was originally in Swedish but dubbed in Dutch.

### A semi-automatic bottom-up approach to obtain vectors of visual semantics from film frames

#### Visual concept recognition model

In order to obtain concept labels per frame we first extracted all frames from the film’s visual stream and converted them to image files. Then, a pretrained commercial deep artificial neural network *Clarifai* ‘*General’* (www.clarifai.com) was used to obtain the concept labels per frame image. Frame images with original RGB colors, 768 × 576 in size were input into the *Clarifai* concept recognition model. A preset dictionary of 5,000 unique concepts was used. The output of the *Clarifai* model contained 20 most likely concept labels per image (= frame) and a probability score per label. A total of 518 unique labels were assigned to frames in our image set.

The output of the visual concept recognition model was then manually corrected. First, we only considered labels with a probability more than 90%, and after making sure that this way we are not losing any unique relevant labels per frame, we discarded the labels with a lower probability. Then, we removed all the labels, which were incorrectly assigned to the images, for example, ‘dog’, ‘piano’, ‘mirror’, ‘battlefield’, ‘zoo’, etc. Then, we removed labels that we deemed irrelevant or difficult to interpret, for example, ‘television’, ‘actor’, ‘abstract’, ‘surreal’, ‘insubstantial’, ‘illustration’, etc. Finally, we restricted the list of all labels to nouns (such as ‘people’, ‘nature’, ‘horse’, ‘food’), adjectives describing a well-defined state or relation ( such as ‘seated’, ‘equestrian’, ‘wooden’) and some adverbs (such as ‘together’, ‘indoors’, ‘outdoors’). We also kept various labels describing action (such as ‘walk’, ‘travel’, ‘dance’, ‘climb’, ‘smile’, etc.). However, most labels referred to objects present in the frame, for example, ‘house’, ‘table’, ‘animal’, ‘rock’ etc. The manual correction procedure resulted in a reduction of the list of unique labels to 129 labels (SI Table S1). Finally, we manually checked that frames did not lack any relevant concept labels from the refined label list.

#### Language model

We used a word embedding model to associate each film frame with a numerical representation that would capture the combined semantics of all the concept labels per frame. For this, we used a pretrained *fastText* model (www.fasttext.cc), which is the extended version of a skip-gram language model, trained by predicting the context of a target word.

We downloaded the semantic vectors that were learned by the *fastText* model when trained on English Wikipedia^40^. The downloaded material contained pairs of words and their corresponding numerical semantic vectors, or word embeddings. We looked up a corresponding vector for each label of each frame. The obtained vectors were of length 300, which means that the semantic space of the *fastText* language model was organized along 300 dimensions, each potentially capturing some relevant language information. Thus, words with similar meanings are represented by vectors with similar values along the 300 dimensions.

Then, per frame, we averaged all numerical semantic vectors (averaging over all labels per frame). This way we were able to obtain one semantic vector of length 300 per frame.

#### Principal component analysis

The principal component analysis was performed on the averaged semantic vectors obtained in the previous step and pursued two goals. First, we aimed to reduce the dimensionality of the semantic vectors. Second, we were interested in a transformation of the semantic space that would uncover the dimensions of the most variance. We suspected that the semantic data used in the present study only captured a small set of the semantic distinctions between all words in the language model because of our focus only on the visually perceived concepts and due to the length and the narrative consistency of the film.

We set the number of principal components to 50 as this transformation provided a considerable reduction in dimensionality of the data while preserving over 99.9% of all variance.

### Interpretation of the extracted semantic vectors

To offer interpretation for the top five principal semantic components we focused on the examples at the minimum and maximum extreme ends per each component. We visualized the frames along with histograms of the concept labels per component.

Then, we performed a post-hoc statistical check by estimating an ordinary least squares fit for the semantic components based on the binary vectors of concept labels. Having observed that the fit was significant (under $p\ll 0.001$, Bonferroni corrected for the number of semantic components) for each of the top five semantic components, per semantic component we ranked the labels according to the regression weight values from most negative to most positive ones.

In addition, we visualized the multidimensional semantic space of 50 components using a 2D projection based on the *t-SNE*^89^ algorithm (SI Figure S2). T-SNE is a dimensionality reduction technique that projects high-dimensional data to a low-dimensional space suitable for visualization (for example, 2D). The algorithm first represents each data point in the high-dimensional space through a conditional probability distribution over its neighbors (*neighbor* *embeddings*). Then it computes the projection to the low-dimensional space that preserves these conditional probabilities (*neighbor* *embeddings*) by minimizing the Kullback–Leibler divergence between the high-dimensional and low-dimensional probability distributions. The *t-SNE* low-dimensional projection is considered one of the optimal techniques for high-dimensional data visualization that preserves relationships between data points at different scales^89^. Here, we used the *scikit-learn*^90^ implementation of the t-SNE technique with default parameters (nearest neighbors = 30, learning rate = 200, metric = squared Euclidian distance, number of iterations = 1,000).

### ECoG experiment

#### Participants and procedures

All participants were admitted for diagnostic procedures with medication-resistant epilepsy. They underwent subdural electrode implantation to determine the source of seizures and test the possibility of surgical removal of the corresponding brain tissue. Research could be conducted between clinical procedures. All patients gave written informed consent to participate in accompanying electrocorticography (ECoG) recordings and gave permission to use their data for scientific research. For participants under 18, the informed consent was obtained from the participant’s parents and/or legal guardian. The study was approved by the Medical Ethical Committee of the Utrecht University Medical Center in accordance with the Declaration of Helsinki (2013).

Thirty-seven patients (age 26 ± 12, 24 females) participated in the film-watching experiment. Thirty patients were implanted with left hemispheric grids. Most patients had left hemisphere as language dominant, based on fMRI, Wada or functional transcranial Doppler sonography tests (Table 1).

**Table 1:** Electrode grid information for all participants.

Patient	N of electrodes	Grid hemisphere	Cortices covered	Handedness	Language dominance
1	96	L	F, T, P	R	L (Wada)
2	112	L	F, M, T, P, O	R	L (fMRI)
3	96	L	F, M, T, P, O	R	L (Wada)
4	104	L	F, M, T, P	R	L (Wada)
5	48	L	F, M, T, P	L	L (fMRI)
6	120	L	F, M, T	R	L (Wada)
7	112	L	F, M, T, P	R	L (fMRI)
8	64	L	F, M, T	R	L (fMRI)
9	112	L	M, T, P, O	R	L (fMRI)
10	64	L	M, T, P	R	L (Wada)
11	96	R	M, T, P, O	L	R (Wada)
12	88	L	T, P, O	R	L (fMRI)
13	112	L	F, T, P	R	R (Wada)
14	120	R	F, M, T	R	L (Wada)
15	96	R	F, T, P, O	L	L (Wada)
16	120	L	F, T, P, O	not available	L (Wada)
17	88	L	F, M	R	L (fMRI)
18	96	L	F, M, T, P, O	L	L (Wada)
19	64	R	T, P, O	R	not assessed
20	64	L	F, M, T, P	R	L (fMRI)
21	88	L	F, M, P	R	L (fMRI)
22	80	L	F, M, T, P	R	L (Wada)
23	128	L	F, M, T, P, O	R	L (fMRI)
24	80	R	F, M, T, P, O	R	L (fMRI)
25	64	L	M, T, P, O	L	L (fMRI)
26	64	R	T, P, O	R	L (Wada)
27	64	L	F, M, T	R	L (fMRI)
28	64	L	F, M, T	R	L (fMRI)
29	48	R	F, M, T	L	R (fMRI)
30	112	L	F, M, T	R	R (fMRI)
31	64	L	F, M, T	R	L (fTCD)
32	64	L	F, M, T, P	R	L (fMRI)
33	120	L	T, P, O	R	L (fMRI)
34	72	L	F, M	L	R (fMRI)
35	72	L	F, M	R	bilateral (fMRI)
36	96	L	F, M, T	R	L (Wada)
37	80	L	F, M, T	R	L (fTCD)

Shown is information about the number of electrodes, grid hemisphere, covered cortices, handedness, and language-dominant hemisphere per patient.

*L* left, *R* right, *F* frontal cortex, *M* motor cortex, *T* temporal cortex, *P* parietal cortex, *O* occipital cortex, *fMRI* functional magnetic resonance imaging, *fTCD* functional transcranial Doppler sonography.

All patients were implanted with clinical electrode grids (2.3 mm exposed diameter, inter-electrode distance 10 mm, between 48 and 128 contact points); one patient had a high-density grid (1.3 mm exposed diameter, inter-electrode distance 3 mm). Almost all patients had temporal grid coverage and most had electrodes in frontal, parietal and motor cortices. The total brain coverage of the patients can be seen in Fig. 3. Patient-specific information about the grid hemisphere, number of electrodes, and cortices covered is summarized in Table 1.

As mentioned above the ECoG data analyzed here came from a film-watching experiment that used a specific experimental design (interleaved blocks of speech and music), as it was originally created for the clinical diagnostic purposes. However, as the stimulus retained a lot of its naturalistic nature, these data were later successfully reused for research purposes targeting auditory processing during watching a film^30,38^. The present work focuses on another aspect of the film stimulus, namely the semantics of the visual narrative.

In the film-watching experiment, each patient was asked to attend to the film displayed on a computer screen (21 inches in diagonal). The stereo sound was delivered through speakers with the volume level adjusted for each patient.

During the experiment ECoG data were acquired with a 128-channel recording system (Micromed, Treviso, Italy) at a sampling rate of 512 Hz filtered at 0.15–134.4 Hz. The film was presented using Presentation (version 18.0, Neurobehavioral Systems Inc) and sound was synchronized with the ECoG recordings.

#### ECoG data processing

All electrodes with noisy or flat signal (visual inspection) were excluded from further analyses. After applying a notch filter for line noise (50 and 100 Hz), common average rereferencing was applied to all clinical grids per patient (and separately for the one high-density grid). Data were transformed to the frequency domain using Gabor wavelet decomposition at 1–120 Hz in 1 Hz bins with decreasing window length (four wavelength full-width at half maximum). Finally, high frequency band (HFB) amplitude was obtained by averaging amplitudes for the 60–120 Hz bins and the resulting time series per electrode were down-sampled to 25 Hz, which corresponded to the frame rate of the film. Electrode locations were coregistered to the anatomical MRI in native space using computer tomography scans^91,92^ and Freesurfer (https://surfer.nmr.mgh.harvard.edu/). The Desikan–Killiany atlas^93^ was used for anatomical labeling of electrodes (closest cortical structure in the radius of 5 mm). All electrode positions were projected to Montreal Neurological Institute space using SPM8 (Welcome Trust Centre for Neuroimaging, University College London).

#### Brain visualization: volume and surface projections

For volume-based visualizations, we used electrode projections to the subject-specific anatomical volume obtained with the electrode localization tool^91^. Then, individual electrode locations were normalized to the MNI space using patient-specific affine transformation matrices obtained with SPM8. For the visualization purposes a 2D Gaussian kernel (FWHM = 8 mm) was applied to the coordinate on the MNI brain volume corresponding to the center of the electrode, so that the projected values (e.g. prediction accuracy) faded out from the center of the electrode toward its borders. All volume-based visualizations were created in MATLAB, version R2018b (https://www.mathworks.com).

For surface-based visualizations we used Freesurfer functions to project the volume-based electrode coordinates to the subject-specific anatomical surface. Then, these coordinates were projected to the subjects’ common Freesurfer space for further visualization on the inflated surface.

However, we noticed that raw projections of the electrode center coordinates resulted in quite patchy visualizations, so we decided to apply some smoothing to all of the cortical overlays based on the electrode values. Because of the often occurring overlaps in electrode grids within and between subjects as well as irregularities of grid placement across subjects, in order to ensure a good result we imposed a regular uniform grid on the common Freesurfer surface and made projections of each individual electrode to the closest point of the regular grid. We used the uniform icosahedron grid of order 5 – *ico5*, distributed with the Freesurfer package. The smoothing was achieved by applying a Gaussian process algorithm^94^ to transform the values of each single subject’s electrodes to the regular grid in the average subject space. Using this approach allowed for a projection of all values to a regular grid while taking into account the cortical distances between electrodes. Values coming from multiple electrodes projected onto the same regular grid coordinate were combined by considering their distances to that coordinate as well. We used the following Gaussian process implementation with an exponential kernel for the electrode distance matrices:

1 $$g={K}_{c}^{T}{\left({K}_{s}+\eta I\right)}^{-1}y$$

2 $${K}_{c}=exp\left(-\frac{{D}_{c}^{2}}{2{\sigma }^{2}}\right)$$

3 $${K}_{s}=exp\left(-\frac{{D}_{s}^{2}}{2{\sigma }^{2}}\right)$$

where ${D}_{s}$ is a matrix of pairwise distances between electrodes of a single subject, ${D}_{c}$ is a matrix of pairwise distance between electrodes of a single subject and the points of the regular grid *ico5* and $\eta$ and $\sigma$ are the free parameters: $\eta$ is a noise parameter and $\sigma$ is the amount of smoothing in the exponential kernel. Vector $y$ represents values to be projected from the single subject electrode space to the regular grid space. The distance matrices ${D}_{s}$ and ${D}_{c}$ are calculated as great circle distances on the average Freesurfer sphere surface:

4 $${d}_{uv}=r \bullet arcos({u}^{T}v)$$

where $r$ is the sphere radius, and vectors $u$ and $v$ are the normalized coordinates of the points on the sphere. In the case of a subject’s electrodes, the vertex corresponding to the location of the center of the electrode is used, and in the case of the regular grid *ico5*, the point on the grid is used.

Both matrices ${D}_{s}$ and ${D}_{c}$ are calculated using single subject electrode coordinates projected to the average Freesurfer sphere with Freesurfer resampling functions.

All surface-based visualizations were produced using Freesurfer visualization tool Freeview (https://surfer.nmr.mgh.harvard.edu/fswiki/FreeviewGuide).

### High accuracy of predicting the neural responses based on the extracted semantic components

#### Neural encoding model based on the semantic components

We used the previously extracted semantic components to predict the neural responses. A ridge linear regression model was employed. The values of the regularization parameter were determined using five-fold nested cross-validation. Pearson correlation between predicted and observed HFB responses in a held-out test set was used to evaluate model performance. The model performance was cross-validated using five-fold cross-validation. The correlation values were averaged across five cross-validation folds and were transformed to *t* values for determining significance^95^. The correlation values reported here were significant at $p<0.001$, Bonferroni corrected for the number of electrodes.

Because of the possible interaction between auditory and visual streams of the short film, we implemented a number of corrections in our model. First, because the auditory stream contained a block design with interleaved blocks of speech and music, we regressed out both the block design and the auditory envelope from both the semantic components and the HFB signal. In case of HFB signal, we first determined the optimal lag for regressing out block design and audio envelope through linear fitting at multiple lags (block design) or best lag of cross-correlation (audio envelope). Both were done independently and separately per each electrode. Cortical maps of regression to the block design and cross-correlation to audio envelope along with the histograms of lags and electrode labels are shown in Supplementary Material (SI Figure S3).

Apart from fitting the regression on semantic components to the residuals of the regressions to block design and audio envelope, we also made sure that the test set per each cross-validation fold contained time points from multiple music and speech blocks. That is the test set of each cross-validation fold was constructed by concatenation of 6 s fragments across multiple music and speech blocks. This was done to avoid block specific effects in testing of each cross-validation fold.

#### Testing various time shifts

Having finalized the details of the linear regression fit, we then performed the fit at multiple time shifts around the stimulus onset to determine the amount of delay in high-level semantic processing of the visual information. Per time shift, we analyzed the average model performance as well as the number of electrodes with a significant fit (at $p<0.001$, Bonferroni corrected for the number of electrodes).

### Relation of the cortical networks to individual semantic components

Next, we aimed to investigate the relationship between brain responses and individual semantic components. In a linear regression model this relationship is reflected in the sign and magnitude of the regression coefficients, or $\beta$-weights. Thus, we focused on the $\beta$-weights of the neural encoding model fitted on the semantic components at the time shift that provided the highest prediction accuracy (~ 320 ms). We selected $\beta$-weights only of those electrodes, whose HFB responses were predicted significantly well by the model. The selected $\beta$-weights were averaged over five cross-validation folds and z-scored over electrodes per semantic component.

Then, an affinity propagation clustering^96^ approach was employed to find groups of electrodes with similar $\beta$-weight profiles across the semantic components. This clustering approach was used due to its non-parametric nature (no requirement to specify the number of clusters beforehand) as well as the ability to identify cluster *exemplars*, or data points representative of the entire cluster. We varied the value of the *preference* parameter but the main set of clusters (Fig. 5) was found for all clustering configurations. The present results are reported for the *preference* value equal to $min\left(A\right)-2$, where $A$ is the affinity matrix. Similarly, we used either Pearson or Spearman correlation coefficient for computing the affinity matrix, and found no significant difference. The reported results were obtained using Pearson correlations.

The described clustering configuration produced 13 clusters, however we only reported clusters that were non-subject-specific, i.e. they contained electrodes from at least one third of all subjects (12/37) and no more than a third of all electrodes in the cluster came from a single subject. Thus, we discarded six clusters: cluster 8 (7 different subjects; 65% electrodes came from a single subject), cluster 9 (7; 36%), cluster 10 (11; 37%), cluster 11 (4; 75%), cluster 12 (14; 42%) and cluster 13 (12; 70%).

For the remaining clusters we reported full cluster profiles containing information about the distribution over subjects, distribution over the cortical regions, the activation time course and the cortical projection map. The distribution over cortical regions was obtained by computing histograms over the cortical labels, associated with the location of the center of each electrode. The activation time courses were calculated as the dot product between the semantic component values and the $\beta$-weights of the cluster exemplar, which is the electrode with the most representative cluster-specific $\beta$-weight profile.

To explore the relationship between each cluster’s activation time course and individual semantic components we applied statistical testing to the frames that were associated with the peaks of the cluster’s activation time course and the frames associated with its dips. In order to assess the statistical significance of peaks and dips in each cluster’s activation time course we shuffled cluster assignments (10,000 times) to obtain a baseline distribution of activation time courses per each cluster. Per cluster, we plotted its activation time course and highlighted the values corresponding to the 2.5th and 97.5th percentiles of the baseline distribution (which corresponds to a two-tailed statistical threshold at p < 0.05). For subsequent analyses relating each cluster’s time course to the semantic components we only considered peaks and dips of the cluster’s activation time course that were outside of the bulk of the baseline distribution. For peaks, we considered frames above the 97.5th percentile, for dips we considered frames below the 2.5th percentile. Then, we selected the frames corresponding to the top 10% of peaks and the bottom 10% of dips in the cluster’s activation time course. Per semantic component, we performed a two-sided Wilcoxon signed rank test to compare values along the semantic component in peaks and dips of the cluster’s activation profile. The significance of the statistic was set at $p<0.001$, Bonferroni corrected for the number of clusters and semantic components. The procedure was repeated for each cluster. The reported values of the statistic were corrected by the amount of variance each semantic component explained by dividing the statistic over the percentage of the explained variance. This was done to correct for the gradual decrease in the magnitude of values along each semantic component due to its decreasing percentage of the explained variance.

### Control for the low-level visual features

As we sought to implement control for the confounding effect of the low-level visual features, we assessed the relationship between low-level visual features, the semantic components and the associated neural responses. First, we assessed the difference in the inner representation of the film frames in low-level features and the semantic components. As low-level features we used raw colored pixel values of the frame images (*pixel*) and Gabor features (*gabor*) configured to model retinotopic responses of complex cells in the early visual cortex of the human brain^97^. Gabor features were extracted from the greyscale pixel values by passing them through a Gabor wavelet pyramid with predefined set of filter sizes and orientations. We followed the filter specifications used in the previous work^97^.

Having extracted *pixel* and *gabor* features we computed the amount of similarity between each of them and the semantic components. For this, we computed pairwise correlations between all film frames using each type of representation: *pixel*, *gabor* and semantic components. Then, we assessed the difference between *pixel* and semantic components as well as *gabor* and semantic components using two-sided Wilcoxon signed rank tests.

Next, we assessed the difference in the prediction accuracy of the neural responses using *pixel*, *gabor* and semantic components. However, both *pixel* and *gabor* feature sets comprised thousands of features (22,500 *pixel* features and 10,920 *gabor* features), whereas the set of the semantic features only contained 50 components, which led to considerable differences in model complexity. To control for this difference, we projected each low-level feature set onto a lower-dimensional space of only 50 components using the principal component analysis, similar to the approach taken in obtaining the semantic features. Then, the ridge linear regression models using either *pixel* or *gabor* 50 principal components were fitted following the same procedures as previously described for the fit on the semantic components. Similarly, for consistency of the comparison, we fixed the temporal shift for these linear models to the best temporal shift reported for the fit using the semantic components—320 ms. The difference in the prediction accuracy between the *pixel* model and the model using the semantic components was assessed using a one-sided Wilcoxon signed rank test on prediction values for all electrodes with significant performance in either model ($p< 0.001$, Bonferroni corrected for the number of electrodes). The difference between the *gabor* model and the model using the semantic components was assessed in the same way.

In addition, we fitted the ridge linear models using either *pixel* or *gabor* features at a temporal shift of 80 ms to examine early visual processing responses.

### Emergence of the visual semantics from the low-level visual features

Next, we assessed the relationship of the semantic components with the visual representations across the object recognition neural network. Because we previously used a commercial model to obtain the labels, we did not have access to its intermediate layer representations. Instead, here we used intermediate representations of another popular object recognition network, called VGG16^50^. VGG16 was pretrained to identify 1,000 object labels in input images. Due to a large network size, we limited our analyses to the representations of all the intermediate pooling layers ($n=5$) of the VGG16 object recognition network. Thus, we passed every film frame through the pretrained VGG16 model and preserved only the representations of the five pooling layers. Due to a large number of frames, per filter of each pooling layer we applied 2D Gaussian smoothing to the frame representations and downsampled them along the image x and y dimensions.

Having obtained the frame representations per pooling layer, we calculated the amount of similarity between them and the frame representations based on the semantic components. This was done by first computing pairwise correlations between all frames per layer, which resulted in $l$ square matrices of size $f\times f$, where $l$ is the number of layers and $f$ is the number of frames. Same was done for the frame representations based on the semantic components. Then, per layer-specific matrix we took all its values in the upper triangle and correlated them with the upper triangle of the matrix based on semantic components. In essence, we performed a simplified version of the representational similarity analysis^98,99^, where the semantic components were the target representation and the pooling VGG16 layers were the candidate representations and Pearson correlation was used as a similarity measure. For the statistical inference about the change in similarity with the semantic components across the pooling layers we performed bootstrapping. This was done by recalculating Pearson correlation between the frame similarity in semantic components and each pooling layer in random samples of 1,000 frames 10,000 times.

Then, we fitted a ridge linear regression (following all the same procedures as previously described) on each of the intermediate VGG16 pooling layers to predict HFB ECoG responses. As in the case of the low-level visual features, we projected each set of the VGG 16 pooling layer features onto a lower-dimensional space of only 50 components using the principal component analysis (original number of features: 87,616 *pool1* features; 41,472 *pool2* features; 43,264 *pool3* features; 18,432 *pool4* features; 25,088 *pool5* features). Similarly, we fixed the temporal shift for these regressions to the best temporal shift reported for the fit using the semantic components—320 ms. The prediction accuracy was cross-validated and projected on the brain volume using the procedures outline above. We also reported scatter plots showing the difference in prediction accuracy between the fit on pooling layers of VGG16 and the fit on the semantic components (Fig. 6c, SI Figure S7). The difference in the prediction accuracy was assessed using one-sided Wilcoxon signed rank tests on prediction values for all electrodes with significant performance in either a layer-specific model or a model using the semantic components ($p< 0.001$, Bonferroni corrected for the number of electrodes).

### Contribution of the language model to the extracted semantic components

Finally, we analyzed the difference in prediction accuracy between the ridge linear model using the semantic components and the ridge linear model using the concept labels for prediction of the associated neural responses. The main difference between the two models was the usage of a language model in construction of the semantic components. In both models we used the manually corrected concept labels. Concept labels were represented as binary categorical vectors with each value in the vector corresponding to a specific label. Per frame, the value of zero corresponded to the absence of the corresponding concept in the frame image and the value of one corresponded to its presence. The ridge linear model was fitted following the same procedure as previously described for the fit on the semantic components except that the regression of the block design and the audio envelope was not applied to the binary label vectors due to the nature of the categorical label data. To make the comparison with the model using the semantic components appropriate, we retrained the linear regression model on the semantic components without regressing out the block design and the audio envelope from the semantic components data as well. Importantly, both models (the one using the binary labels and the other using the semantic components) were fitted on the brain data regressed to the audio envelope and block design at an optimal lag per electrode (see the procedures above) to keep the effects of the audio-visual interactions to the minimum. The prediction accuracies of both models were compared at the fixed temporal shift of 320 ms.

The difference in the prediction accuracy between the two models was assessed using a one-sided Wilcoxon signed rank test on prediction values for all electrodes with significant performance in either model ($p<0.001$, Bonferroni corrected for the number of electrodes).

## Supplementary information

Supplementary information.

## Data Availability

Custom code supporting the results of the current study is available at https://github.com/Immiora/semantic_encoding_ecog_clarifai_fasttext. The ECoG data have not been deposited in a public repository due to the restrictions on public sharing of the patients’ data but are available from the corresponding author on request.
