# Supplementary material for: Cortical network responses map onto data-driven features that capture visual semantics of movie fragments
Source: Sci Rep. 2020 Jul 21;10:12077. doi: 10.1038/s41598-020-68853-y (PMC7374611; doi:10.1038/s41598-020-68853-y)
Supplement: Supplementary file 1 — Supplementary information. [file 41598_2020_68853_MOESM1_ESM.docx]

| UNIVERSITY MEDICAL CENTER UTRECHT |
| --- |
| Cortical network responses map onto data-driven features that capture visual semantics of movie fragments |
|  |
|  |
| **Julia Berezutskaya¹ ², Zachary V. Freudenburg¹, Luca Ambrogioni², Umut Güçlü²,**  **Marcel A. J. van Gerven² and Nick F. Ramsey¹** |
|  |

| Brain Center, Department of Neurology and Neurosurgery,  University Medical Center Utrecht, Heidelberglaan 100, 3584 CX,Utrecht, The Netherlands¹  Donders Institute for Brain, Cognition and Behaviour, Radboud University, Montessorilaan 3, 6525 HR,Nijmegen, The Netherlands²  Corresponding author: [y.berezutskaya@umcutrecht.nl](mailto:y.berezutskaya@umcutrecht.nl) |
| --- |
|  |

**Supplementary material**

**Supplementary table 1.** List of all concept labels used in the study.

| 1 | acrobat | 34 | dark | 67 | happiness | 100 | reptile |
| --- | --- | --- | --- | --- | --- | --- | --- |
| 2 | acrobatics | 35 | daylight | 68 | harness | 101 | restaurant |
| 3 | adolescent | 36 | domestic | 69 | head | 102 | road |
| 4 | adult | 37 | door | 70 | home | 103 | rock |
| 5 | agility | 38 | drink | 71 | horse | 104 | rodent |
| 6 | agriculture | 39 | enjoyment | 72 | house | 105 | roof |
| 7 | amphibian | 40 | environment | 73 | indoors | 106 | room |
| 8 | animal | 41 | equestrian | 74 | leaf | 107 | run |
| 9 | architecture | 42 | equine | 75 | lunch | 108 | rural |
| 10 | ballerina | 43 | exploration | 76 | mammal | 109 | seated |
| 11 | ballet | 44 | exterior | 77 | man | 110 | sky |
| 12 | band | 45 | eye | 78 | mane | 111 | smile |
| 13 | barn | 46 | facade | 79 | meal | 112 | sport |
| 14 | beard | 47 | face | 80 | mouse | 113 | stallion |
| 15 | bench | 48 | facial | 81 | mouth | 114 | storm |
| 16 | body | 49 | family | 82 | music | 115 | street |
| 17 | boy | 50 | farm | 83 | mustache | 116 | summer |
| 18 | brick | 51 | farming | 84 | nature | 117 | table |
| 19 | building | 52 | fence | 85 | nose | 118 | together |
| 20 | candle | 53 | field | 86 | one | 119 | togetherness |
| 21 | cap | 54 | food | 87 | outdoors | 120 | transportation |
| 22 | cavalry | 55 | fox | 88 | owl | 121 | travel |
| 23 | ceiling | 56 | friendship | 89 | park | 122 | tree |
| 24 | chair | 57 | frog | 90 | pasture | 123 | walk |
| 25 | child | 58 | fun | 91 | pavement | 124 | wet |
| 26 | climb | 59 | furniture | 92 | people | 125 | wildlife |
| 27 | concert | 60 | girl | 93 | performance | 126 | window |
| 28 | countryside | 61 | glue | 94 | person | 127 | woman |
| 29 | couple | 62 | grass | 95 | picnic | 128 | wooden |
| 30 | crowd | 63 | ground | 96 | pony | 129 | young |
| 31 | dance | 64 | group | 97 | portrait |  |  |
| 32 | dancer | 65 | hair | 98 | rain |  |  |
| 33 | dancing | 66 | hand | 99 | rainforest | |  |


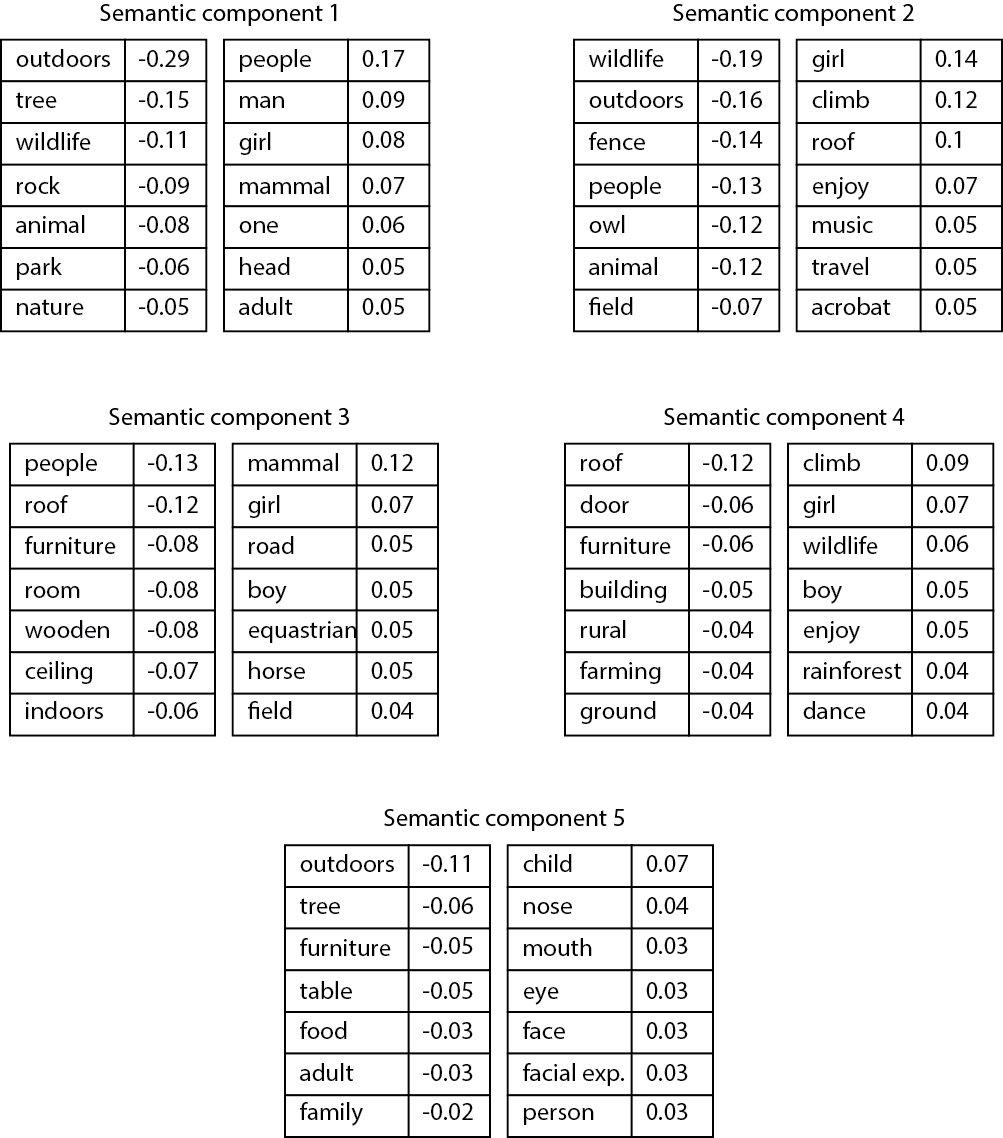


**Supplementary figure 1.** Labels with largest absolute values of $\beta$-weights in the linear regression fitting the concept labels to the five semantic components. The largest negative weights are displayed in the left column per component and the largest positive weights are displayed in the right column.


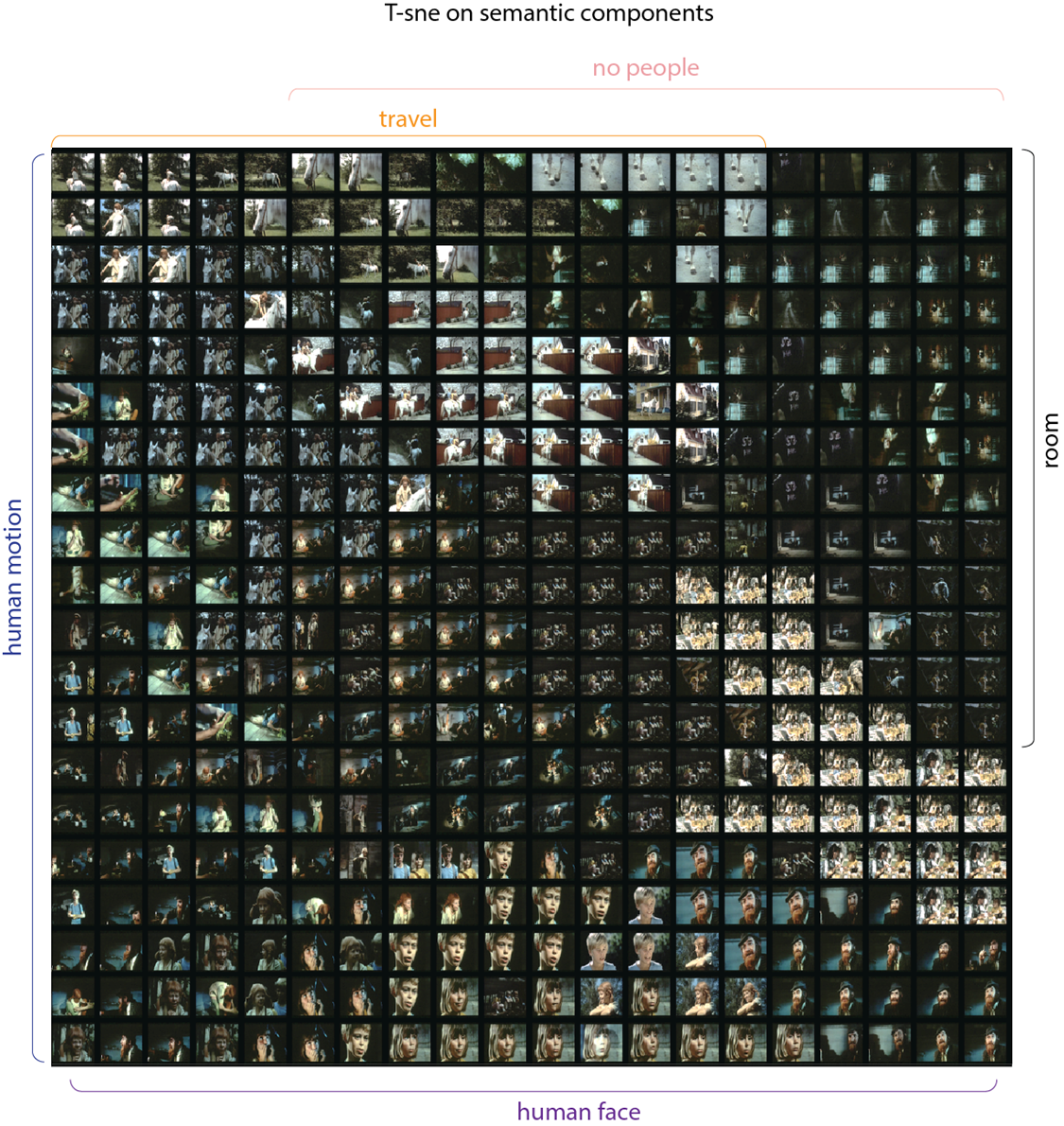


**Supplementary figure 2.** T-SNE visualization of the frames based on their semantic component representation. This visualization procedure projects a multidimensional space of semantic components (d=50) to a two-dimensional representation while preserving the distances between individual frames as much as possible (see Methods for details). This projection offers a possibility to notice clusters of frames organized in distinct semantic groups: human faces at the bottom, human motion and object interaction on the left, closed spaces and no people present in the top right corner, travel and human movement in the top left corner.


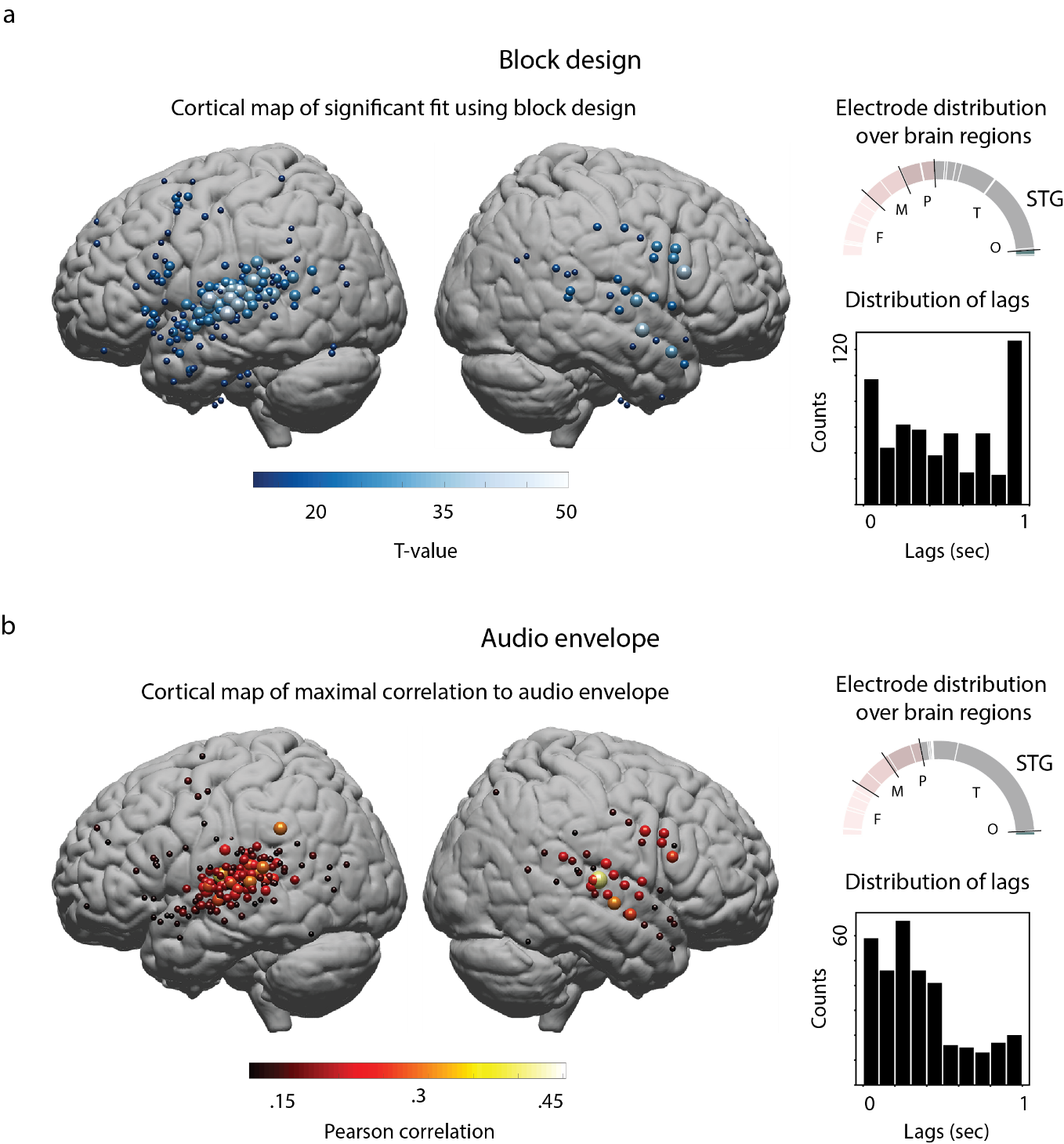


**Supplementary figure 3.** Control for audio envelope and block design in the stimulus. **(a)** Ordinary least squares fit of the HFB responses based on the block design in the film soundtrack. The fit was performed per each lag within 1 second after the audio onset to account for the neural delay during sensory processing. The displayed *t*-values correspond to the fit at the best time lag (at $p<.001$, Bonferroni corrected for the number of electrodes and number of lags). Left panel shows the cortical distribution of electrodes with a significant fit at their individual best fitted lag. Top right panel shows the distribution over the cortical areas for all the electrodes with a significant fit. Different areas are color-coded: F: frontal, M: motor, P: parietal, T: temporal, O: occipital regions. The section of the chart containing electrodes from the superior temporal gyrus is additionally labeled (STG). Bottom right panel shows a distribution of best lags for the electrodes with a significant fit. **(b)** Cross-correlation to the audio envelope. Left panel shows the highest correlation within 1 second after the audio onset (at $p<.001$, Bonferroni corrected for the number of electrodes and lags). Right panel shows distribution over the cortical regions and time lags.


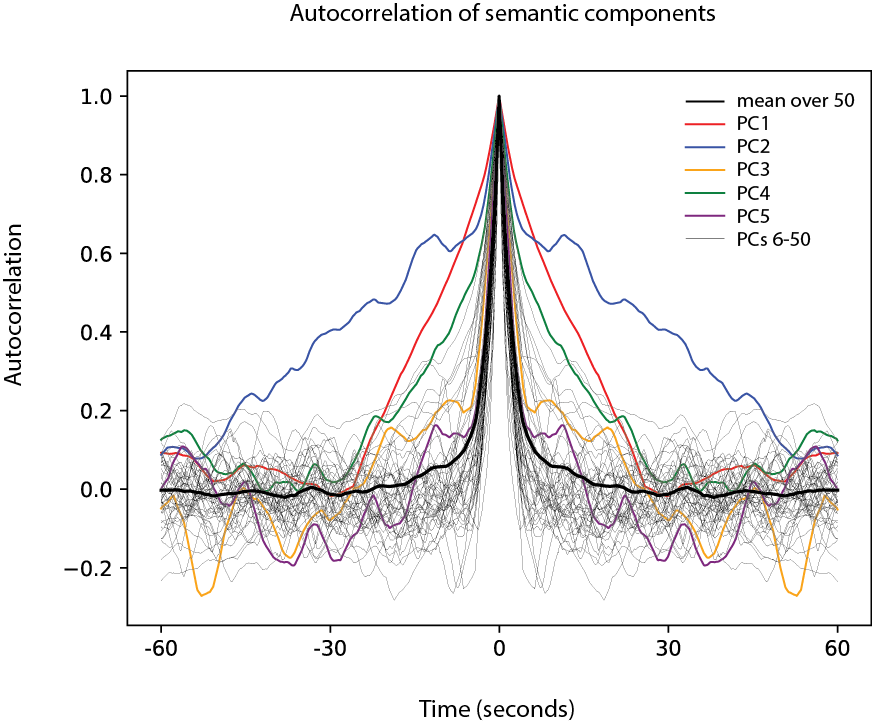


**Supplementary figure 4.** Autocorrelation of the semantic components. Colored lines refer to the autocorrelation of the top five semantic components (same color scheme as in Figures 2 and 6). Bold black line shows mean autocorrelation across all 50 semantic components. Thin black lines show autocorrelation for the remaining 45 semantic components.


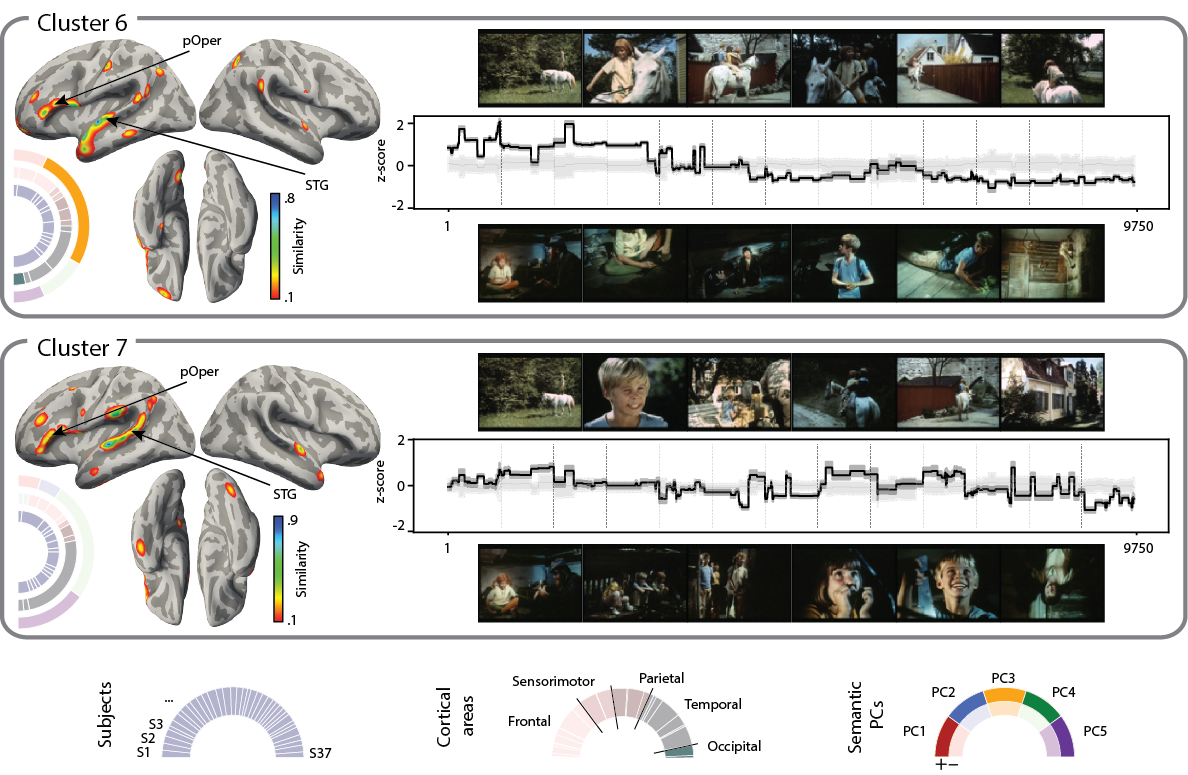


**Supplementary figure 5.** Remaining clusters capturing distributed functional cortical networks associated with each individual semantic component. The display follows the same format as in **Figure 5** of the main text.

**
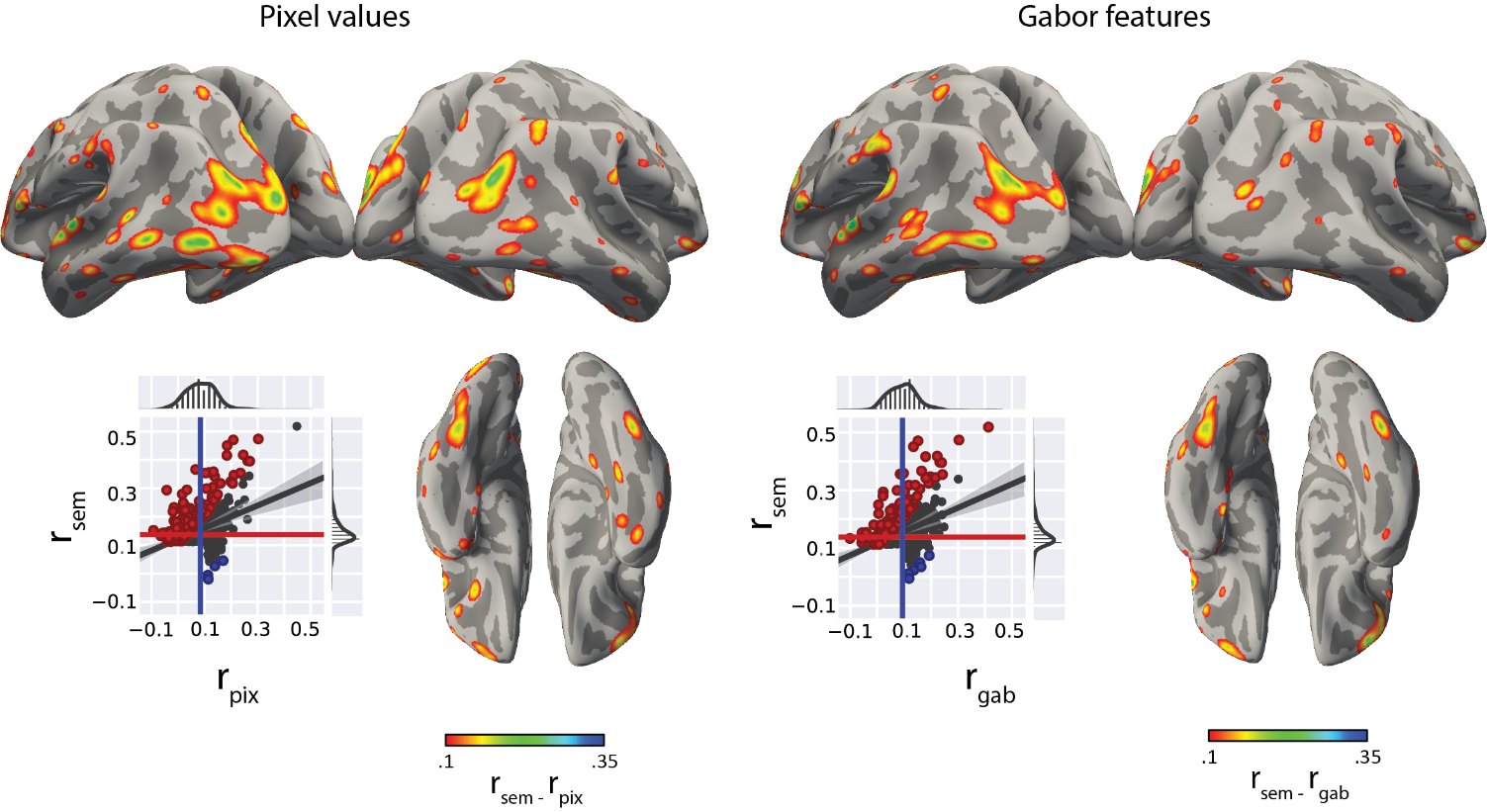
**

**Supplementary figure 6.** Comparison with low-level visual features, including colored pixel values as well as Gabor features that model responses of complex cells in primary visual cortex. Ridge linear regression to the HFB responses was fitted for each additional feature set the same way as when using the semantic components as input features. The results are shown for the models fitted at the temporal shift of 320 ms with respect to the stimulus onset. Shown are the cortical maps of difference in the prediction accuracy between the fit using low-level features and the semantic components. In addition, scatter plots comparing the prediction accuracy between each low-level visual feature set and the semantic components are displayed. For both the cortical maps and the scatter plots, only the electrodes with a significant fit with either model ($sem$and $pix$or $sem$and$gab$) at $p<.001$, Bonferroni corrected for the number of electrodes were used. Scatter plots also show the median prediction accuracy (over all significant electrodes) per each model: the model using 50 semantic components (red line) and a control low-level visual feature model (blue line). Histograms of the prediction accuracy over all significant electrodes are also shown per model.

**
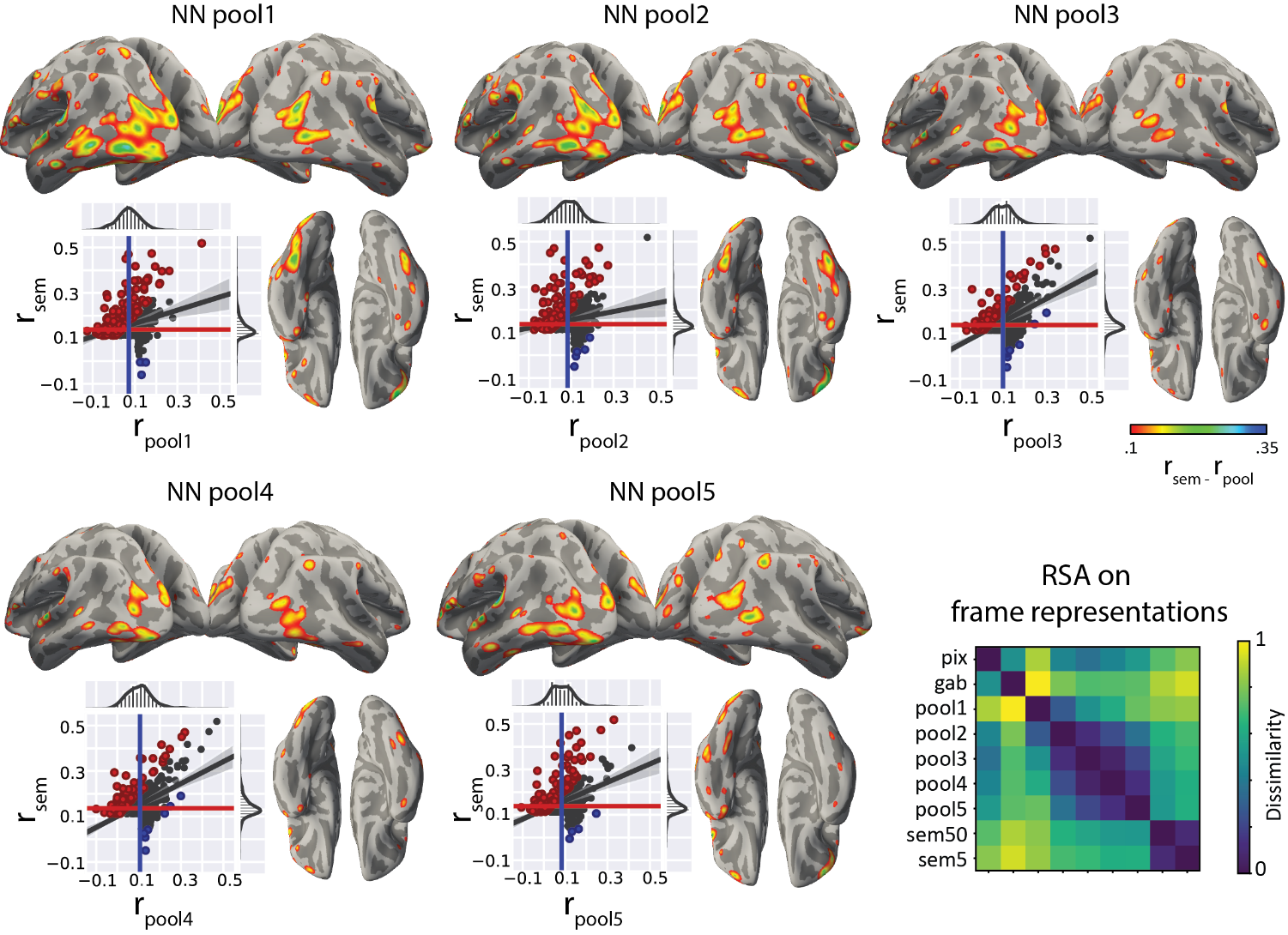
**

**Supplementary figure 7.** Comparison with features of the intermediate layers of the automatic visual object recognition model (pooling layers of VGG16). Ridge linear regression to the HFB responses was fitted per each additional feature set the same way as when using the semantic components as input features. The results are shown for the models fitted at the temporal shift of 320 ms with respect to the stimulus onset. Shown are the cortical maps of difference in the prediction accuracy between the fit using pooling layers of VGG16 and the semantic components. In addition, scatter plots comparing the prediction accuracy between a feature set from each of the pooling layers and the 50 semantic components are displayed. For both the cortical maps and the scatter plots, only the electrodes with a significant fit with either model in each comparison ($sem$and $pool$) at $p<.001$, Bonferroni corrected for the number of electrodes, were used. Scatter plots also show the median prediction accuracy (over all significant electrodes) per each model: the model using 50 semantic components (red line) and a control model using features from the pooling layers (blue line). Histograms of the prediction accuracy over all significant electrodes are also shown per model. Bottom left plot shows the dissimilarity matrix, comparing frame representations across various visual features (low-level and intermediate layers of the object recognition model) and the semantic components (top five or all 50 semantic components).
